# Drinking alcohol is associated with variation in the human oral microbiome in a large study of American adults

**DOI:** 10.1186/s40168-018-0448-x

**Published:** 2018-04-24

**Authors:** Xiaozhou Fan, Brandilyn A. Peters, Eric J. Jacobs, Susan M. Gapstur, Mark P. Purdue, Neal D. Freedman, Alexander V. Alekseyenko, Jing Wu, Liying Yang, Zhiheng Pei, Richard B. Hayes, Jiyoung Ahn

**Affiliations:** 1 Department of Population Health, NYU School of Medicine, 650 First Avenue, Room 518, New York, NY 10016 USA; 2 Epidemiology Research Program, American Cancer Society, 250 Williams Street NW, Atlanta, GA 30303 USA; 3 Division of Cancer Epidemiology and Genetics, National Cancer Institute, 9609 Medical Center Drive, Rockville, MD 20850 USA; 4 Biomedical Informatics Center, Departments of Public Health Sciences and Oral Health Sciences, Program for Human Microbiome Research, Medical University of South Carolina, Charleston, SC 29425 USA; 5 Department of Medicine, NYU School of Medicine, 423 East 23rd St, New York, NY 10010 USA; 6 NYU Laura and Isaac Perlmutter Cancer Institute, 522 First Avenue, New York, NY 10016 USA; 7 Department of Pathology, NYU School of Medicine, 550 First Avenue, New York, NY 10016 USA; 8 Department of Veterans Affairs New York Harbor Healthcare System, New York, NY 10010 USA

**Keywords:** Oral microbiome, 16s rRNA genes, Alcohol consumption, Large population-based study

## Abstract

**Background:**

Dysbiosis of the oral microbiome can lead to local oral disease and potentially to cancers of the head, neck, and digestive tract. However, little is known regarding exogenous factors contributing to such microbial imbalance.

**Results:**

We examined the impact of alcohol consumption on the oral microbiome in a cross-sectional study of 1044 US adults. Bacterial 16S rRNA genes from oral wash samples were amplified, sequenced, and assigned to bacterial taxa. We tested the association of alcohol drinking level (non-drinker, moderate drinker, or heavy drinker) and type (liquor, beer, or wine) with overall microbial composition and individual taxon abundance. The diversity of oral microbiota and overall bacterial profiles differed between heavy drinkers and non-drinkers (α-diversity richness *p* = 0.0059 and β-diversity unweighted UniFrac *p* = 0.0036), and abundance of commensal order *Lactobacillales* tends to be decreased with higher alcohol consumption (fold changes = 0.89 and 0.94 for heavy and moderate drinkers, *p* trend = 0.005 [*q* = 0.064]). Additionally, certain genera were enriched in subjects with higher alcohol consumption, including *Actinomyces*, *Leptotrichia*, *Cardiobacterium*, and *Neisseria*; some of these genera contain oral pathogens, while *Neisseria* can synthesize the human carcinogen acetaldehyde from ethanol. Wine drinkers may differ from non-drinkers in microbial diversity and profiles (α-diversity richness *p* = 0.048 and β-diversity unweighted UniFrac *p* = 0.059) after controlling for drinking amount, while liquor and beer drinkers did not. All significant differences between drinkers and non-drinkers remained after exclusion of current smokers.

**Conclusions:**

Our results, from a large human study of alcohol consumption and the oral microbiome, indicate that alcohol consumption, and heavy drinking in particular, may influence the oral microbiome composition. These findings may have implications for better understanding the potential role that oral bacteria play in alcohol-related diseases.

**Electronic supplementary material:**

The online version of this article (10.1186/s40168-018-0448-x) contains supplementary material, which is available to authorized users.

## Background

More than 700 different bacterial species and a range of other microorganisms (archaea, fungi, and viruses) colonize the human oral cavity, known collectively as the oral microbiome [1, 2]. Oral microbiota play important roles in human health, including in immune response, carcinogen metabolism, and nutrient digestion [3–5]. Evidence indicates that oral microbiota dysbiosis is related to local oral diseases, such as periodontitis and dental caries [6] and potentially to systemic diseases, including gastrointestinal cancers [7, 8] and cardiovascular disease [9]. However, little is known regarding exogenous exposures that cause dysbiosis of the oral microbiota.

We hypothesized that alcohol drinking habits are associated with changes in the oral microbial community. Alcohol intake may impact the oral microbiota in several ways: by direct cytotoxic effects on bacteria [10], by disturbing saliva-bacterium interactions [11–14], and by providing ethanol as a substrate for bacterial metabolism [4]. Several previous studies, in both animals and humans, have also observed effects of alcohol consumption on oral bacteria. Animal studies showed that diet containing 20% ethanol increases colonization by *Streptococcus mutans* [15], a caries-related bacterium, and dramatically decreases the number of detectable bacterial species in the oral biofilms of rats [16]. Similarly, the association of excessive drinking (> 3 drinks per day) with poor oral health was observed in a population study [17]. In small-scale human studies, drinking at least one glass of red wine per day was associated with reduced species richness and reduction of certain anaerobic bacteria in sub- and supra-gingival plaque [18], while excessive co-use of tobacco and alcohol was associated with reduced species richness and decreased abundance of *Neisseria*, *Aggregatibacter*, and *Fusobacteria*, in oral mucosa biofilms [19]*.* Aside from direct effects, alcohol may indirectly impact the oral microbiota through disturbing the host defense system [20–23], subsequently resulting in host-mediated periodontitis [24, 25]. Large population-based studies have consistently demonstrated that alcohol consumption is associated with increased risk of periodontal disease in a dose-dependent fashion [24, 26]. Evidence shows that the oral microbiome is closely tied to oral health status [27, 28]. Despite this evidence suggesting an impact of alcohol on the oral microbiome, no study has comprehensively investigated the relationship of the oral microbiome to alcohol drinking habits in terms of drinking amount and types of alcoholic beverages consumed.

We tested the relationship of level and types of alcoholic beverages, with the oral microbiome in 1044 individuals from two large US national cohorts. The oral microbiome was characterized by bacterial 16S rRNA gene sequencing. Comparisons of overall community composition and taxon abundance were conducted across groups with respect to drinking status.

## Methods

### Study population

Participants were drawn from the American Cancer Society (ACS) Cancer Prevention Study II (CPS II) Nutrition cohort [29] and the National Cancer Institute (NCI) Prostate, Lung, Colorectal, and Ovarian Cancer Screening Trial (PLCO) cohort [30]. As previously described [31], subjects included in the present analyses were originally selected from the CPS II and PLCO cohorts as cases or controls for collaborative nested case-control studies of the oral microbiome in relation to head and neck cancer and pancreatic cancer. Oral wash samples were collected by mail from 70,004 CPS II Nutrition cohort participants between 2000 and 2002 and in the PLCO control arm (*n* = 77,445) at recruitment from 1993 to 2001. After excluding participants without information on alcohol consumption status, 458 participants from CPS II (*n* = 169 from the head and neck study and *n* = 289 from the pancreas study) and 586 participants from PLCO (*n* = 231 from the head and neck study and *n* = 355 from the pancreas study) were included in the current study. All participants provided informed consent and all protocols were approved by the New York University School of Medicine Institutional Review Board.

### Alcohol consumption and covariate assessment

Comprehensive demographic and lifestyle information was collected by baseline and follow-up questionnaires in the PLCO and CPS II Nutrition cohorts. Detailed information on alcohol consumption over the past year, including frequency of consumption, serving size, and type of alcoholic beverages consumed (wine, beer, and liquor), was ascertained via questionnaires that were most close in time to oral wash sample collection for both cohorts. The 137-item Food Frequency Questionnaire (FFQ) and Diet History Questionnaire (DHQ) were used in PLCO and CPS II Nutrition cohorts, respectively [32, 33]. According to the drinking level definition of the *Dietary Guidelines for Americans* 2010 [34], we defined moderate drinkers as > 0 but ≤ 1 drinks per day, on average, for women, and > 0 but ≤ 2 drinks per day, on average, for men. Women and men who had greater than one or two drinks per day, respectively, were considered heavy drinkers. This definition considers the gender differences in blood concentration of ethanol after drinking [35], which is equal to salivary ethanol for up to 5 h after consumption [36]. Drinkers exclusively consuming wine, beer, or liquor were defined as wine drinkers, beer drinkers, and liquor drinkers, respectively.

### Oral microbiota characterization using 16S rRNA gene amplification and sequencing

Participants in both cohorts were asked to swish vigorously with 10 mL Scope mouthwash (P&G) for 30 s and then to expectorate into a specimen tube. Samples were shipped to each cohort’s biorepository, pelleted, and stored at − 80 °C until use [30, 37]. Bacterial genomic DNA was extracted from the samples using the MoBio PowerSoil DNA Isolation Kit (Carlsbad, CA), with the bead-beating method in the MoBio Powerlyzer instrument. As reported previously [38], 16S rRNA gene sequencing on the extracted DNA was performed. 16S rRNA amplicon libraries were generated using primers incorporating FLX Titanium adapters and a sample barcode sequence, allowing unidirectional sequencing covering variable regions V3 to V4 (Primers: 347F- 5′GGAGGCAGCAGTAAGGAAT-3′ and 803R- 5′CTACCGGGGTATCTAATCC-3′). Five nanograms of genomic DNA was used as the template in 25 uL PCR reaction buffer for 16S rRNA amplicon preparation. Cycling conditions were one cycle of 94 °C for 3 min, followed by 25 cycles of 94 °C for 15 s, 52 °C for 45 s, and 72 °C for 1 min followed by a final extension of 72 °C for 8 min. The generated amplicons were then purified using Agencourt AMPure XP kit (Beckman Coulter, CA). Purified amplicons were quantified by fluorometry using the Quant-iT PicoGreen dsDNA Assay Kit (Invitrogen, CA). Equimolar amounts (10^7^ molecules/uL) of purified amplicons were pooled for sequencing. Pyrosequencing (Roche 454 GS FLX Titanium) was carried out according to the manufacturer’s instructions [39].

### Upstream sequence analysis of microbiome data

16S rRNA gene amplicon sequences were processed and analyzed using the QIIME pipeline [40]. Multiplexed libraries were deconvoluted based on the barcodes assigned to each sample. Poor-quality sequences were excluded using the default parameters of the QIIME script *split_libraries.py* (minimum average quality score = 25, minimum/maximum sequence length = 200/1000 base pairs, no ambiguous base calls, and no mismatches allowed in the primer sequence). From 1044 pre-diagnostic oral wash samples, we obtained 11,395,395 high-quality 16S rRNA gene sequence reads (mean 10,915 [SD = 3049] sequences per sample), with similar number of reads in all cohorts [31]. Filtered sequence reads were clustered into operational taxonomic units (OTUs) and subsequently assigned to taxa by using the Human Oral Microbiome Database (HOMD) pre-defined taxonomy map of reference sequences with ≥ 97% identity [41]. Summary of sequence reads per sample which were assigned to HOMD reference is shown in Additional file 1: Table S1.

### Quality control

Blinded positive quality control (QC) specimens were used across all sequencing batches. We previously reported good agreement of microbiome parameters in replicates from these QC subjects [31]. Negative control samples (with Scope mouthwash only) were used to detect possible bacteria in Scope and reagent, as well as environment contamination in all sequencing batches. No DNA was detected from the negative control samples. We further conducted an independent experiment which compared the oral microbiota in paired mouthwash samples and immediately frozen whole saliva samples from 10 healthy subjects. The results showed that oral microbial profiles in mouthwash samples were similar to the profiles in saliva samples (Additional file 1: Figure S1). Thus, the mouthwash samples are suitable to test the hypothesis in this study.

### Statistical analysis

The effect of drinking level was assessed by comparing subjects in each drinking category (moderate, heavy) to non-drinkers. For trend tests, drinking level was treated as a continuous variable by assigning the numbers 0, 1, and 2 to non-, moderate, and heavy drinkers, respectively. The effect of drinking type (wine, beer, or liquor) was examined by modeling the three dichotomous variables of any consumption of wine, beer or liquor, while additionally adjusting for multiple drinking types using cross-product terms (e.g., formula to test effect of wine drinking: ~any_wine + any_beer + any_liquor + any_wine*any_beer + any_wine*any_liquor). To control for the effects of potential confounders, all models were adjusted for age, sex, race, BMI category (normal weight, overweight, obese), smoking status (never, former, current), education level (no college, some college, college graduate), and study (CPSII-a, CPSII-b, PLCO-a, PLCO-b); drinking type models were additionally adjusted for drinking amount (grams of ethanol per day).

We assessed α-diversity (within-subject diversity) using numbers of observed species (richness) and the inverse Simpson’s Index (evenness). These α-diversity indices were calculated in 500 iterations of rarefied OTU tables with the minimum sequencing depth of 1325 among all study subjects. The average over the iterations was taken for each participant. Linear regression with covariate adjustment was used to examine the difference of α-diversity indices among drinking groups. β-diversity (between-subject diversity) was assessed using unweighted and weighted UniFrac distance matrices accounting for both presence or absence of observed organisms and their abundance, respectively [42]. We performed Permutational Multivariate Analysis of Variance (PERMANOVA; adonis function, vegan package, *R*) [43] and partial constrained analysis of principal coordinates (partial CAP; “capscale” function, vegan package, *R*) [44] to examine statistically and visually whether bacterial community profiles differed by drinking level or type. Unlike the commonly used unconstrained principal coordinate analysis (PCoA) representation of UniFrac distances, partial CAP displays the variation attributable to alcohol consumption with covariate adjustment. We also conducted pairwise comparisons among drinking groups for each of the first three coordinates in unconstrained PCoA, using the Kruskal-Wallis post-hoc test (Dunn’s test). The community type analysis of the oral wash samples was performed with Dirichlet multinomial mixture (DMM) model using counts of sequencing reads at the genus level [45].

Operational taxonomic units (OTUs) were classified into 13 phyla, 23 classes, 37 orders, 76 families, 227 genera, and 341 species, according to their alignment with the HOMD reference database. We used the “DESeq” function within the DESeq2 package [46] in *R* to test for differentially abundant taxa by drinking level and types of alcoholic beverages, at each taxonomic level. This function models raw counts using a negative binomial distribution and adjusts internally for “size factors” which normalize for differences in sequencing depth between samples. We analyzed taxa within the major oral phyla (carried by more than 90% of study subjects). Additionally, we filtered to include only taxa with greater than 2 sequences in at least 100 participants using *R* command in DESeq “rowSums(counts(OTU.deseq) > 2) ≥ 100” to remove low-count taxa for the phylum through species level analysis. This resulted in inclusion of 5 phyla, 9 classes, 15 orders, 30 families, 96 genera, and 95 species in the analysis. DESeq2 default outlier replacement and filtering of count outliers were turned off. Taxa models with a maximum Cook’s distance > 10 were removed prior to *p* value adjustment for the false discovery rate (FDR). To account for the multiple comparisons at each taxonomic level, we considered an FDR-adjusted *p* value (*q* value) less than 0.10 as significant. Pearson’s linear correlation was used to explore the correlation among the log-transformed DESeq2-normalized taxa abundance. All statistical tests were two-sided, and all statistical analyses were carried out using *R* version 3.2.1.

To examine if smoking, future cancer case/control status, or oral health status (periodontal disease and caries) has confounding effects on the observed alcohol-microbiome associations, we conducted several sensitivity analyses: (a) excluding current smokers, (b) adjusting for future case/control status in models, (c) excluding subjects who had detectable periodontal pathogens *Porphyromonas gingivalis* and/or *Aggregatibacter actinomycetemcomitans* in their oral samples (surrogate markers for periodontal disease [47]), and (d) stratified analysis by the median abundance of *Streptococcus mutans* (surrogate marker for dental caries [48]). The latter two analyses used surrogate markers of oral disease as we lacked information on oral disease in our study.

## Results

### Participant characteristics

Of the 1044 participants in this study, 25.9% (*n* = 270) were non-drinkers, 58.8% (*n* = 614) were moderate drinkers, and 15.3% (*n* = 160) were heavy drinkers. The study participants were predominantly above middle age (mean 67.7, range 55–87) and White (95%), with age and race distributions not differing significantly by drinking level. The alcohol drinking groups had higher percentages of men and smokers and tended to be leaner and more educated than the non-drinker group (Table 1). Among alcohol drinkers, 13.0% (*n* = 101) were wine-only drinkers, 5.0% (*n* = 39) were beer-only drinkers, and 3.4% (*n* = 26) were liquor-only drinkers (Table 2).

**Table 1:** Demographic characteristics of the study participants

	Non-drinkers (*n* = 270)		Moderate drinkers* (*n* = 614)		Heavy drinkers* (*n* = 160)		*p* value^†^
	*N*	%	*N*	%	*N*	%	
Age^‡^		68.3 ± 7.0		67.6 ± 7.4		66.8 ± 6.8	0.092
Gender							
Male	150	55.6	412	67.1	103	64.4	
Female	120	44.4	202	32.9	57	35.6	0.0044
Race							
White	255	94.4	581	94.6	157	98.1	
Non-White	15	5.6	33	5.4	3	1.9	0.16
BMI, kg/m^2^							
< 25	87	32.2	226	36.8	70	43.7	
25–< 30	114	42.2	273	44.5	64	40.0	
≥ 30	69	25.6	115	18.7	26	16.3	0.039
Education							
High school or less	96	33.6	188	30.6	39	24.4	0.014
Some college	93	34.4	178	29.0	54	33.8	
College graduate or higher	81	30.0	248	40.4	67	41.9	
Smoking status							
Never	157	58.1	266	43.3	38	23.8	
Former	92	34.1	301	49.0	98	61.3	
Current	21	7.8	47	7.7	24	15.0	< 0.0001

.According to the Dietary Guidelines for Americans, moderate drinking was defined as > 0 to 1 drink per day for women and > 0 to 2 drinks per day for men; more than 1 drink per day for women and 2 drinks per day for men was defined as heavy drinking

^†^*p* values were from chi-square test or ANOVA

^‡^Mean and standard deviation were calculated

**Table 2:** Types of alcoholic beverages consumed by drinkers. Subjects who ever consumed alcohol were included in the table (*n* = 774)

	Number of drinkers in each drinking type		Mean of pure ethanol consumption
	*N*	%	gram/day
Any wine drinkers	645	83.3	15.5
Wine only^†^	101	13.0	3.8
Wine and other types	544	70.3	17.7
Any beer drinkers	565	73.0	19.9
Beer only^†^	39	5.0	20.7
Beer and other types	526	68.0	19.8
Any liquor drinkers	545	70.4	19.0
Liquor only^†^	26	3.4	7.0
Liquor and other types	519	67.0	19.6

^†^Wine, beer, or liquor drinkers exclusively consumed each type of beverages respectively

### Drinking level analysis

**Fig. 1:**
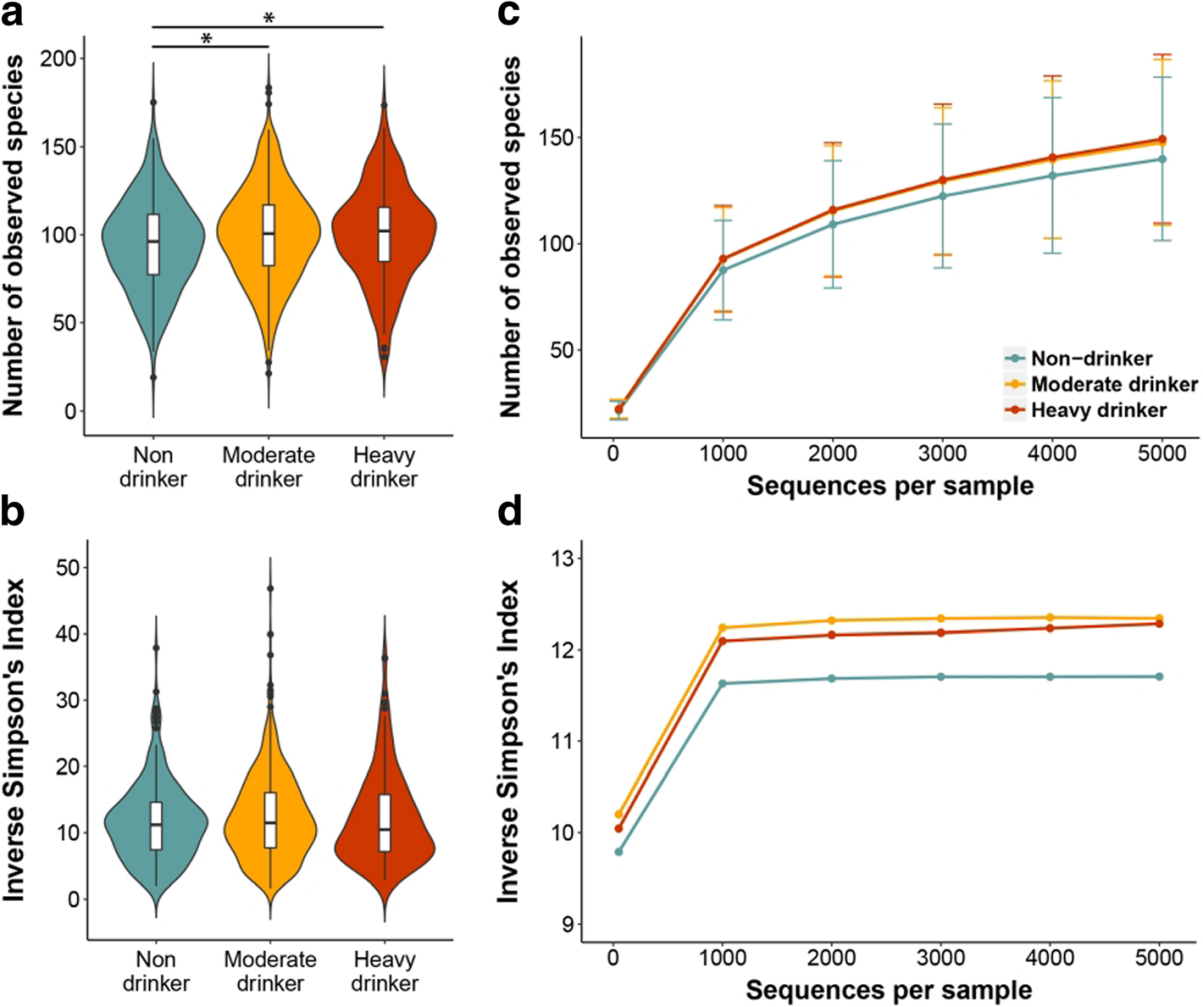
Richness and evenness of oral microbiome by alcohol drinking levels. **a**, **b** Violin plots of the number of observed species (richness) and inverse Simpson’s Index (evenness) in non-drinker, moderate drinker (MD) and heavy drinker (HD) groups. These indices were calculated for 500 iterations of rarefied OTU table with minimum sequencing depth of 1325 among all study subjects, and the average over the iterations was taken for each participant. Plotted are median, interquartile ranges, and the probability density of the indices at different values. Mean values of the richness in non-drinker, MD, and HD groups were 94.6, 100.1, and 100.3, respectively; mean values of the inverse Simpson’s Index in each group were 11.6, 12.3, and 12.1. One star (*) indicates *p* < 0.05 in linear regression model. **c**, **d** Rarefaction curves of number of observed species and inverse Simpson’s Index according to the number of reads per sample in non-drinkers, MD, and HD

**Fig. 2:**
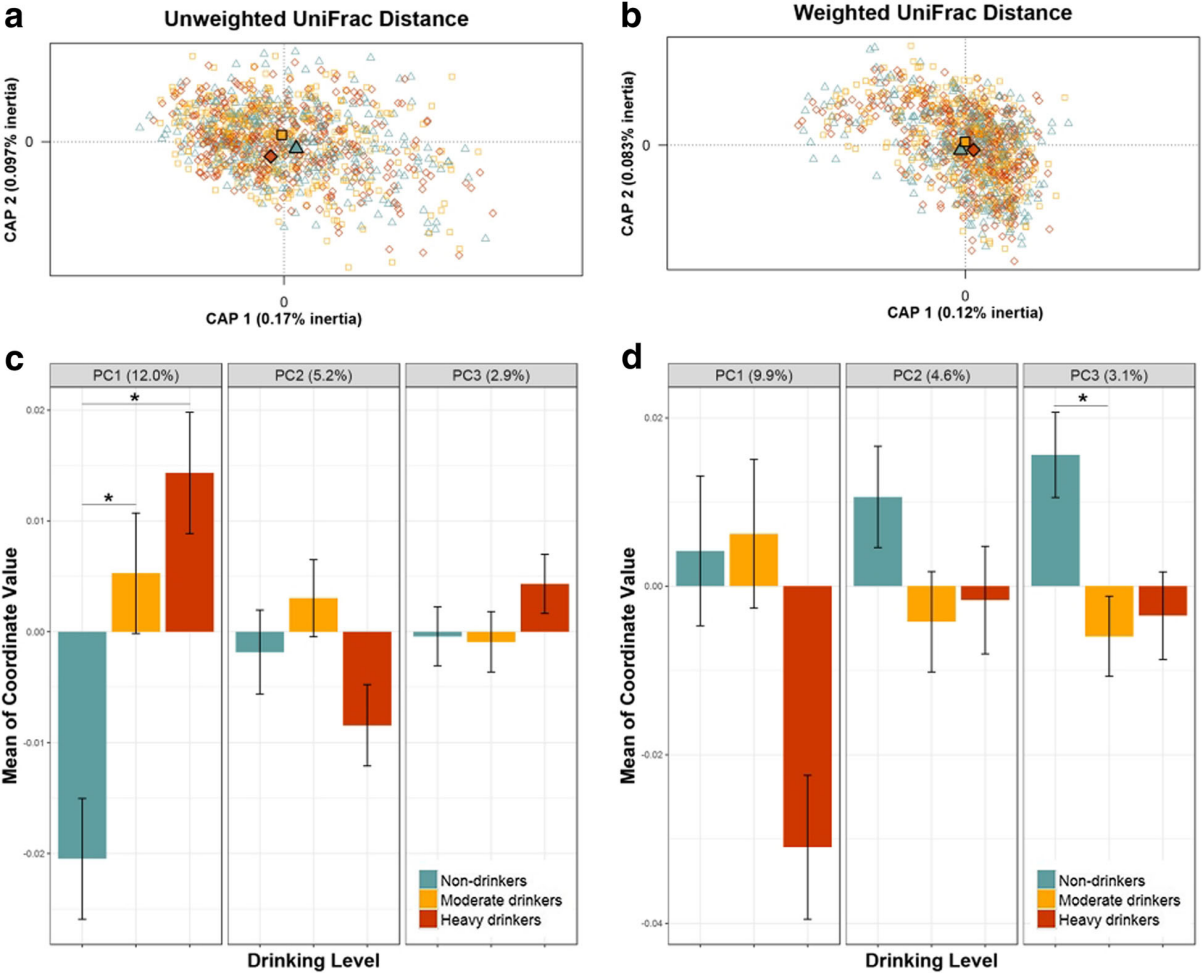
Partial constrained analysis of principal coordinates (CAP) and Principal Coordinate Analysis (PCoA) plots. **a**, **b** CAP plots using unweighted and weighted UniFrac phylogenetic distance matrices in all study participants. Drinking level was the constraining variable; age, race, gender, BMI, education, smoking status, and study were treated as partial variables. Filled shapes indicate centroids for each group. **c**, **d** Bar plots showing the means of the first, second, and third coordinates of PCoA for each drinking level using unweighted and weighted UniFrac phylogenetic distance matrices in all study participants. One star (*) indicates *p* < 0.05 in the Kruskal-Wallis post hoc test (Dunn’s test)

We first examined the overall microbial composition according to drinking level. In the α-diversity analysis, richness (Fig. 1a, c) increased in heavy drinkers (HD) and moderate drinkers (MD) with statistical significance (mean_HD_ = 100.3, *p* from linear regression = 0.0059 and mean_MD_ = 100.1, *p* = 0.0073) relative to non-drinkers (mean_non-drinker_ = 94.6). These differences in richness remained significant after further adjustment for case/control status (*p* = 0.0051 for HD and 0.0072 for MD). Evenness tended to be higher in drinkers compared to non-drinkers (Fig. 1b, d); however, the differences were not statistically significant (*p* = 0.092 and 0.062 for HD and MD, respectively). When assessing β-diversity according to the unweighted UniFrac distance, partial CAP revealed that HD and MD separated from non-drinkers on the first CAP axis, based on position of the group centroids (Fig. 2a). Similarly, HD and MD differed from non-drinkers in the first principal coordinate in PCoA (*p* = 0.029 and 0.038 respectively) (Fig. 2c). In PERMANOVA analysis of the unweighted UniFrac distance, HD significantly differed from non-drinkers after controlling for covariates (*p* = 0.0036), while MD did not (*p* = 0.22). The difference between HD and non-drinkers in PERMANOVA analysis persisted after further adjustment for case/control status (*p* = 0.0050). When assessing β-diversity according to the weighted UniFrac distance, HD, MD, and non-drinkers clustered together in partial CAP plot (Fig. 2b) and showed no differences in the first three principal coordinates in PCoA, with the exception of the third coordinate comparing MD and non-drinkers (*p* = 0.018) (Fig. 2d). In PERMANOVA analysis of the weighted UniFrac distance, neither HD nor MD were found to be different from non-drinkers (*p* = 0.22 and 0.24 for HD and MD, respectively). The differences in the microbial community composition according to alcohol drinking were confirmed by the results of community type classification (Additional file 1: Figure S2).

**Fig. 3:**
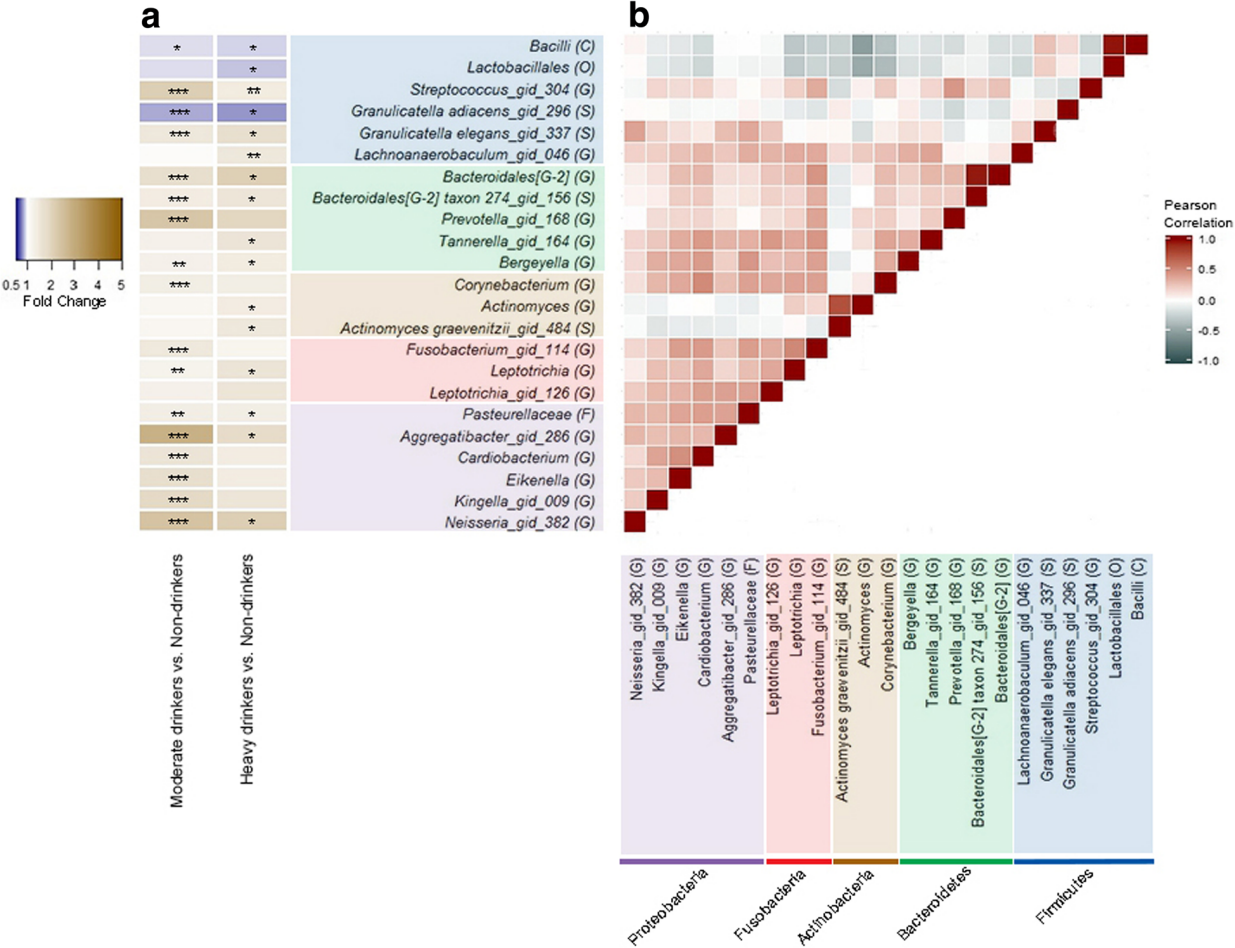
Heatmap of fold changes and the correlations of the taxa related to alcohol drinking level. **a** Fold change for moderate drinkers (*N* = 614) and heavy drinkers (*N* = 160) compared to non-drinkers (*N* = 270) was estimated by DESeq function, adjusting for age, sex, race, BMI, education, smoking status, and study. One star (*) indicates FDR-adjusted *q* < 0.10, two stars (**) indicate *q* < 0.05, and three stars (***) indicate *q* < 0.01, in the DESeq2 analysis. **b** Pearson’s linear correlation matrix of the selected taxa. For correlation analysis, counts were normalized for DESeq2 size factors and log2 transformed after adding a pseudocount of 1. Strong positive correlations are indicated by dark red and strong negative correlations by dark slate-gray. Color coding of taxa names represents the phylum to which each taxa belongs, as follows: *Firmicutes* (blue), *Bacteroidetes* (green), *Actinobacteria* (brown), *Fusobacteria* (red), and *Proteobacteria* (purple)

Associations between drinking level and the abundance of specific oral taxa were examined using negative binomial generalized linear models [46] (Table 3 and Fig. 3a). Abundance of class *Bacilli* in phylum *Firmicutes* was decreased with higher drinking levels (fold changes [FCs] = 0.92 and 0.94 for HD and MD, FDR-adjusted *q* trend = 0.050), as was its major order *Lactobacillales* (FCs = 0.89 and 0.94 for HD and MD, q = 0.064). Low-abundance genera *Streptococcus* and *Lachnoanaerobaculum* in *Firmicutes* were increased with higher drinking levels. Taxa in other phyla were also enriched with higher drinking level, including the genus *Actinomyces* (FCs = 1.41 and 1.15 for HD and MD, *q* = 0.041) and its constituent species *A. graevenitzzi* (FCs = 1.53 and 1.17 for HD and MD, *q* = 0.088). *Leptotrichia* (FCs = 1.61 and 1.24 for HD and MD, *q* = 0.0027) and low-abundance genera *Cardiobacterium* (FCs = 1.41 and 1.61 for HD and MD, *q* = 0.041) and *Neisseria* (FCs = 2.12 and 2.42 for HD and MD, *q* = 0.027) were increased in both heavy and moderate drinkers, although read counts tended to be low for these bacteria (Additional file 1: Figure S3). Additional taxa that were differentially abundant by drinking level are shown in Table 3 and Fig. 3a. The correlation matrix of the taxa associated with drinking level indicated that class *Bacilli* and order *Lactobacillales*, which were decreased with higher drinking level, generally had negative correlations with other taxa that were increased with higher drinking level (Fig. 3b). Results were not altered by further adjustment for case/control status.

**Table 3:** Taxa related to alcohol drinking level in all study participants. The association between taxonomic abundance and alcohol drinking level was detected by DESeq function, adjusted for age, sex, race, BMI, education, smoking status, and study

Taxon Class;Order;Family;Genus;Species	Mean counts^†^			Fold change (95% CI)			
	Non-drinkers	Moderate drinkers	Heavy drinkers	Moderate vs. non-drinkers	Heavy vs. non-drinkers	*p* trend^‡^	*q* trend^§^
Phylum *Firmicutes*							
*Bacilli*	5986.51	5578.79	5459.46	0.94 (0.89, 0.98)	0.92 (0.86, 0.98)	0.0055	0.050
*Bacilli;Lactobacillales*	5687.89	5340.36	5111.72	0.94 (0.88, 1.00)	0.89 (0.82, 0.97)	0.0050	0.064
*Bacilli;Lactobacillaes;Streptococcaceae;Streptococcus_gid_304*	37.39	181.57	58.55	2.13 (1.66, 2.74)	1.44 (1.03, 2.00)	< 0.001	0.015
*Bacilli;Lactobacillales;Carnobacteriaceae;Granulicatella;Adiacens_gid_296*	133.08	110.70	105.93	0.84 (0.76, 0.93)	0.80 (0.70, 0.92)	< 0.001	0.057
*Bacilli;Lactobacillales;Carnobacteriaceae;Granulicatella;Elegans_gid_337*	15.86	23.52	22.88	1.54 (1.16, 2.04)	1.74 (1.22, 2.48)	0.0013	0.057
*Clostridia;Clostridiales;Lachnospiraceae[14];Lachnoanaerobaculum_gid_046*	16.1	16.45	25.01	1.05 (0.86, 1.28)	1.52 (1.16, 1.99)	0.0063	0.042
Phylum *Bacteroidetes*							
*Bacteroides;Bacteroidales;Bacteroidales[F-2];Bacteroidales[G-2]*^||^	3.31	7.05	6.94	1.74 (1.30, 2.33)	2.08 (1.43, 3.03)	< 0.0001	0.0027
*Bacteroides;Bacteroidales;Bacteroidales[F-2];Bacteroidales[G-2];taxon 274_gid_156*	1.64	2.43	2.59	1.42 (1.09, 1.83)	1.56 (1.13, 2.17)	0.0043	0.094
*Bacteroides;Bacteroidales;Porphyromonadaceae;Tannerella_gid_164*	2.28	3.00	3.61	1.28 (0.98, 1.67)	1.60 (1.13, 2.27)	0.0070	0.042
*Bacteroides;Bacteroidales;Prevotellaceae;Prevotella_gid_168*	1.73	43.72	1.98	2.31 (1.64, 3.25)	1.91 (1.24, 2.94)	< 0.001	0.0099
*Flavobacterial;Flavobacteriales;Flavobacteriaceae;Bergeyella*	3.62	4.62	5.29	1.29 (1.09, 1.54)	1.46 (1.15, 1.84)	< 0.001	0.014
Phylum *Actinobacteria*							
*Actinobacteria;Actinomycetale;Actinomycetaceae;Actinomyces*	103.79	123.63	131.63	1.15 (0.97, 1.37)	1.41 (1.11, 1.78)	0.0047	0.041
*Actinobacteria;Actinomycetale;Actinomycetaceae;Actinomyces;Graevenitzii_gid_484*	37.25	47.59	55.77	1.17 (0.95, 1.45)	1.53 (1.16, 2.02)	0.0030	0.088
*Actinobacteria;Actinomycetales;Corynebacteriaceae;Corynebacterium*^¶^	21.93	28.95	29.51	1.35 (1.12, 1.63)	1.36 (1.06, 1.76)	0.0064	0.042
Phylum *Fusobacteria*							
*Fusobacteria;Fusobacteriales;Fusobacteriaceae;Fusobacterium_gid_114*	184.52	369.96	229.27	1.48 (1.24, 1.78)	1.22 (0.95, 1.56)	0.018	0.090
*Fusobacteria;Fusobacteriales;Leptotrichiacefaae;Leptotrichia*	70.25	94.78	157.05	1.24 (1.05, 1.48)	1.61 (1.27, 2.03)	< 0.0001	0.0027
*Fusobacteria;Fusobacteriales;Leptotrichiaceae;Leptotrichia_gid_126*	7.97	10.31	12.10	1.30 (0.99, 1.72)	1.58 (1.10, 2.27)	0.010	0.055
Phylum *Proteobacteria*							
*Grammaproteobacteria;Pasteurellales;Pasteurellaceae*	138.73	194.42	191.54	1.36 (1.12, 1.66)	1.37 (1.07, 1.76)	0.0064	0.052
*Gammaproteobacteria;Pasteurellales;Pasteurellaceae;Aggregatibacter_gid_286*	3.21	10.94	5.63	2.83 (1.93, 4.15)	1.81 (1.13, 2.89)	0.0024	0.027
*Betaproteobacteria;Neisseriales;Neisseriaceae;Eikenella*	1.33	2.20	1.75	1.71 (1.33, 2.19)	1.39 (1.00, 1.93)	0.0099	0.055
*Betaproteobacteria;Neisseriales;Neisseriaceae;Kingella_gid_009*	4.19	8.35	6.12	1.85 (1.38, 2.49)	1.55 (1.06, 2.27)	0.0057	0.042
*Betaproteobacteria;Neisseriales;Neisseriaceae;Neisseria_gid_382*	1.90	5.20	5.55	2.42 (1.55, 3.75)	2.12 (1.27, 3.54)	0.0024	0.027
Grammaproteobacteria;Cardiobacteriales;Cardiobacteriaceae;Cardiobacterium**	5.41	7.47	7.04	1.60 (1.27, 2.01)	1.41 (1.04, 1.92)	0.0047	0.041

^†^Sequence read counts were normalized by dividing raw counts by DESeq size factors

^‡^Nominal *p* values from trend tests. In trend test, alcohol drinking was treated as a continuous variable by assigning the numbers 0, 1, and 2 to non-, moderate, and heavy drinkers, respectively. All taxa with an FDR-adjusted *q* < 0.10 are included in the table. See Additional file 1: Table S1 for mean counts and comparisons stratified by cohort

^§^FDR-adjusted *p* value. FDR adjustment was conducted at each taxonomic level (i.e., class, genus) separately

^||^Association was significant up to family *Bacteroidales[F-2]*, because this genus is the single constituent member of its family

^¶^Association was significant up to family *Corynebacteriaceae*, because this genus is the single constituent member of its family

**Association was significant up to order *Cardiobacteriales*, because this genus is the single constituent member of its order

We examined the homogeneity of our results across two independent cohorts. The partial CAP plots indicated a similar trend of HD separated from non-drinkers on the first CAP axis in both cohorts (Additional file 1: Figure S4a–d), which was further supported by the PERMANOVA analysis showing that HD differed from non-drinkers according to the unweighted UniFrac distance in both CPS II and PLCO cohorts (*p* = 0.097 and *p* = 0.064 respectively). Most of the taxa associated with drinking level showed similar trends in both cohorts (Additional file 1: Figure S3 and Table S2), with the exception of family *Bacteroidales[F-2]*, its constituent genus *Bacteroidales[G-2]*, and genus *Corynebacterium*, which all were shown to increase significantly with drinking level in the PLCO, but not CPS II, cohorts.

In a sensitivity analysis to determine potential influences of smoking and oral disease on study results, the observed alcohol-microbiota associations (i.e., in α-diversity, β-diversity, and taxon abundance) were unchanged after excluding current smokers or subjects carrying periodontal pathogens (Additional file 1: Table S3). Additionally, similar trends for alcohol-microbiota associations were observed in those with low or high abundance of *S*. *mutans*, a marker of dental caries (Additional file 1: Table S3).

### Drinking type analysis

**Fig. 4:**
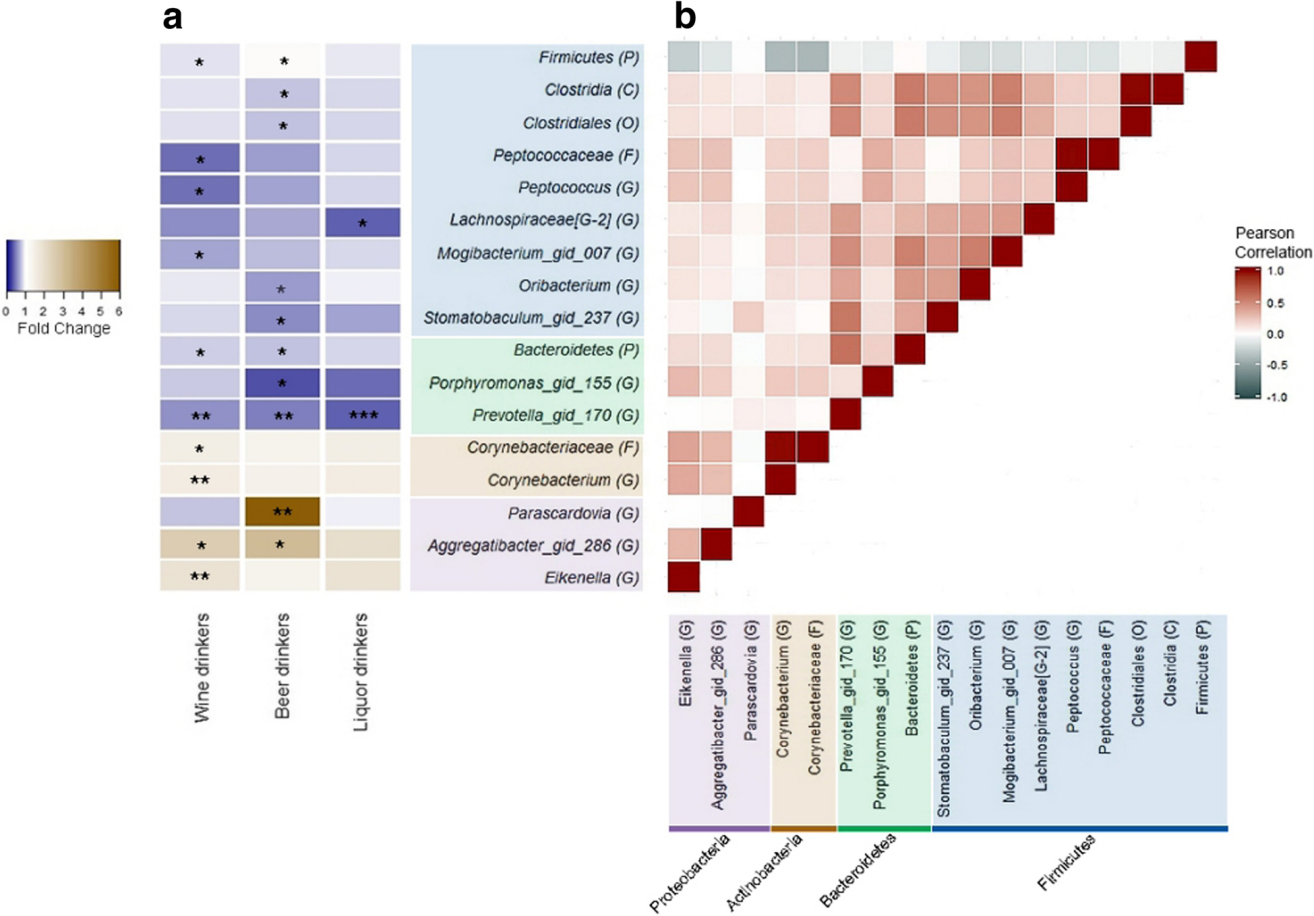
Heatmap of fold changes and the correlations of the taxa related to alcohol drinking type. **a** Fold change for exclusive wine drinkers (*N* = 101), beer drinkers (*N* = 39), and liquor drinkers (*N* = 26) compared to non-drinking group was estimated by DESeq function, adjusting for age, sex, race, BMI, education, smoking status, drinking amount, and study. One star (*) indicates FDR-adjusted *q* < 0.10, two stars (**) indicate *q* < 0.05, and three stars (***) indicates *q* < 0.01, in the DESeq2 analysis. **b** Pearson’s linear correlation matrix of the selected taxa. For correlation analysis, counts were normalized for DESeq2 size factors and log2 transformed after adding a pseudocount of 1. Strong positive correlations are indicated by dark red and strong negative correlations by dark slate-gray. Color coding of taxa names represents the phylum to which each taxa belongs, as follows: *Firmicutes* (blue), *Bacteroidetes* (green), *Actinobacteria* (brown), *Fusobacteria* (red), and *Proteobacteria* (purple)

We next investigated the overall microbial composition and taxon abundances for exclusive wine (*n* = 101), beer (*n* = 39), and liquor (*n* = 26) drinkers, relative to non-drinkers, after controlling for drinking amount. In the α-diversity analysis (Additional file 1: Figure S5), beer drinkers and liquor drinkers did not differ from non-drinkers in richness (*p* from linear regression = 0.87 and 0.91 for beer and liquor drinkers, respectively), while wine drinkers (mean = 100.9, *p* = 0.048) had increased richness compared to non-drinkers (mean = 94.6). Significance remained after further adjustment for case/control status (*p* = 0.048) and exclusion of current smokers (*p* = 0.053). Similarly, in covariate-adjusted PERMANOVA analysis of the unweighted UniFrac distance, wine drinkers differed from non-drinkers with marginal significance (*p* = 0.059), but liquor drinkers or beer drinkers did not (*p* = 0.73 and *p* = 0.59). Evenness (Additional file 1: Figure S5) and β-diversity estimated by weighted UniFrac distance did not reveal any differences by drinking type. Compared to non-drinkers, wine drinking was associated with decreased abundance of genus *Peptococcus* (FC = 0.45, *q* = 0.073); beer drinking was associated with decreased abundance of *Porphyromonas* (FC = 0.32, *q* = 0.071) and increased abundance of genus *Parascardovia* (FC = 5.87, *q* = 0.016); and liquor drinking was associated with decreased abundance of *Lachnospiraceae[G-2]* (FC = 0.39, *q* = 0.090) (Fig. 4 & Additional file 1: Table S4). Other taxa associated with wine, beer, or liquor drinking were in genera *Corynebacterium*, *Prevotella*, and *Aggregatibacter* and *Eikenella*, which had been identified as also associated with drinking level (Fig. 4 and Additional file 1: Table S4).

## Discussion

In this first large comprehensive human study, we observed that overall microbiome community composition in the oral cavity differed by drinking level, with consistent results across two independent cohorts and after exclusion of current smokers. Drinkers had decreased abundance of order *Lactobacillales*, the major order in the *Firmicutes* phylum. Other taxa, some of which are potentially pathogenic, were enriched with higher alcohol consumption. Additionally, wine drinkers may have a shifted microbiome community composition relative to non-drinkers. These results suggest that alcohol drinking and beverage type may influence the oral microbiota.

We studied the oral microbiome in mouthwash samples, which contain bacteria shed from adhering microbial communities on various oral sites, including tooth surfaces, gingival crevices, tongue dorsum, and buccal mucosa [49–51]. We found an increase in within-subject diversity (richness) and qualitative variation [42] in microbial community profiles (unweighted UniFrac) by drinking level. The observed increased diversity and altered profiles in drinkers may be due to direct effects of alcohol or may reflect poor oral health conditions in drinkers. Alcohol intake has been associated with risk of periodontal diseases and caries [17, 24, 26]. Additionally, poor oral health conditions, including higher plaque index, presence of decayed teeth, gingival bleeding, and deeper periodontal pockets, have been associated with higher phylogenetic diversity of salivary microbiota [27]. In a sensitivity analysis, we used periodontal pathogens (*P*. *gingivalis* and *A*. *actinomycetemcomitans*) and the abundance of *S*. *mutans* in saliva as surrogate markers for periodontitis and dental caries, respectively, and major findings remained unchanged. Thus, it is possible that alcohol consumption has a direct effect on oral bacteria composition independently of oral disease.

Our results indicate that alcohol consumption is associated with decreased abundance of *Lactobacillales*, a dominant order in class *Bacilli* and phylum *Firmicutes*. Bacteria in the order *Lactobacillales* (also named lactic acid bacteria or LAB)*,* produce lactic acid as an end-product of carbohydrate fermentation [52] and are among the most common microbes employed as probiotics [53]. *Firmicutes* and *Lactobacillales* abundance have also been shown to decrease in the intestines of mice fed with ethanol chronically [54, 55], while increases were observed in potentially pathogenic bacteria, *Proteobacteria* and *Actinobacteria*, in response to ethanol feeding [54]. While the mechanism for ethanol-induced decreases in *Lactobacillales* are unclear, depletion of *Lactobacillales* may promote growth of alkaline-tolerant bacteria [54], which may explain the inverse correlations of *Lactobacillales* with the other taxa associated with alcohol drinking. In support of this, probiotic treatment of ethanol-fed mice with *Lactobacillus rhamnosus GG* decreased luminal pH and prevented ethanol-related pathogenic increases in the gut microbiome [54]. Evidence shows that *Lactobacillales* have a beneficial effect on oral health [56]. Some *Lactobacillales* can reduce the risk of caries development [57], possibly through competitive exclusion and displacement of pathogens, or by production of antibacterial compounds [58–60]. Additionally, oral *Lactobacilli* species can suppress the growth of periodontal pathogens [61], and oral intake of some *Lactobacillales* can promote reduction of gingival inflammation [62]. Thus, the alcohol-related depletion of beneficial commensal *Lactobacillales* may lead to further oral health inflammation-related disturbances.

We also identified some taxa enriched in drinkers. Ethanol may indirectly increase certain bacterial taxa by decreasing *Lactobacillales* thereby increasing pH, as mentioned above, or by inhibiting the antimicrobial properties of saliva and by disturbing the host-microbial balance. Both acute and chronic ethanol exposure can lead to functional changes in saliva, including decreased flow rate and impaired output of total protein, amylase, and electrolytes [12, 14]. Additionally, alcohol could impair neutrophil function (contributing to bacterial overgrowth and increased bacterial penetration) [20], reduce monocyte production of inflammatory cytokines (allowing for microbial proliferation) [21], and have adverse effects on teeth (stimulate bone resorption and suppress bone formation) [22] and the periodontium [23]. These detrimental effects of alcohol on host defense potentially lead to periodontitis [24]. Among the taxa enriched in drinkers, the genera *Aggregatibacter*, *Actinomyces*, *Kingella*, *Leptotrichia*, *Cardiobacterium*, *Bacteroidales[G-2]*, and *Prevotella* may contain human pathogenic and cardiogenic pathogens. *Aggregatibacter*, a genus in the *Pasteurellaceae* family of the *Proteobacteria* phylum, is related to periodontal disease [63–66] and infective endocarditis [67, 68], as are species in *Actinomyces*, a genus from the *Actinobacteria* phylum [69, 70], and *Kingella*, a genus from the *Proteobacteria* phylum [68, 71, 72]. *Leptotrichia* is one of the two major genera in phylum *Fusobacteria*, which tends to cause disease in the presence of local or general pre-disposing factors and has been reported to be involved in various human infections [73]. The only two species in genus *Cardiobacterium*, *C*. *hominis and C*. *valvarum*, both can cause endocarditis [74–76]. The constituent species *oral taxon 274* in *Bacteroidales[G-2]* was shown to be more prevalent in subgingival sites in periodontitis subjects than in healthy subjects [77]. Species of genus *Prevotella* are part of the indigenous microbiota of mucous membranes from oral cavity, while some black-pigmented *Prevotella*, for instance *P. intermedia* and *P. melaninogenica*, associate with periodontal disease [78]. Thus, the changes in the composition of oral microbiome related to alcohol consumption may potentially contribute to dental caries, periodontal diseases, and other health consequences.

In addition to these compositional changes related to alcohol use, alcohol consumption may result in the bacterial production from ethanol of acetaldehyde, a World Health Organization (WHO) group 1 human carcinogen [79]. Acetaldehyde is toxic, mutagenic, and carcinogenic in human cells and animal models [80–83]. Certain bacteria are involved in producing acetaldehyde from ethanol, while others can beneficially metabolize acetaldehyde to less toxic compounds. *Neisseria* is mainly a non-pathogenic genus in the oral cavity, but it has extremely high alcohol dehydrogenase (ADH) activity and produces significant amounts of acetaldehyde from ethanol [84]. The increased abundance of *Neisseria* with alcohol drinking that we have observed is consistent with the ADH activity of *Neisseria* and is supported by another human study [84]. Conversely, strains of gastrointestinal *Lactobacillus*, decreased in alcohol consumers in our study, show a high capacity to metabolize acetaldehyde to less toxic forms [85]. Thus, ethanol-related increase in abundance of *Neisseria*, which produces acetaldehyde, and decrease in abundance of LAB, involved in acetaldehyde degradation, suggest that oral bacteria may play a role in alcohol-related carcinogenesis through acetaldehyde.

Analyses by beverage type revealed that wine drinkers may have a higher richness and different microbial profiles from non-drinkers, with decreased abundance of phyla *Bacteroidetes* and *Firmicutes*, and family *Peptostreptococcaceae*. These findings are consistent with previous in vivo and in vitro studies investigating the antimicrobial properties of wine, showing that wine drinkers had decreased abundance of certain species in sub- and supra-gingival plaques, including species in *Peptostreptococcaceae* [18, 86]. The results on microbial richness in these studies are inconsistent with our findings, which may relate to different sample types used (subgingival plaque vs. mouthwash samples). Beer has a relatively low alcohol concentration but a complex chemical composition [87], containing a greater abundance of proteins, B type vitamins, fiber, minerals, antioxidants, and varied flavors than other alcoholic beverage types [88], which potentially may have differential effects on the growth of oral bacteria. Due to the high concentration of ethanol in liquor, the bacterial variations associated with liquor drinking may only more closely represent the effects of pure ethanol. Bacterial taxa similarly altered relative to non-drinkers in wine, beer, and liquor drinkers (e.g., *Prevotella_gid_170*) may simply reflect an effect of drinking alcohol vs. not drinking. Because our study had limited numbers of subjects who exclusively consumed beer, wine, or liquor, further study is required to disentangle the differential effect of each type of alcoholic beverage on oral microbial composition.

Our study has several strengths. First, the use of 16S rRNA gene sequencing for microbiome analysis allowed us to comprehensively study overall bacterial community composition and specific oral taxon abundances. Second, our very large sample size provided excellent statistical power to detect variation in the oral microbiome with alcohol drinking and allowed us to confirm our findings in multiple independent study groups. Finally, the detailed information on alcohol drinking amount and type allowed us to classify alcohol intake in detail, and the detailed demographic and lifestyle information allowed us to adjust for potential confounding factors. A limitation of our study is that it is observational, limiting our ability to establish a causal relationship. A trial where moderate or heavy drinkers were randomized to continue or stop drinking for a long enough time to influence the oral microbiome could provide more definitive information. Additionally, a few enriched bacteria in drinkers has overall low abundance. Further, the majority of this study population was White and above middle age, limiting the generalizability of our findings to other races and age groups. We lacked information on the oral health status of the study participants, though sensitivity analysis using bacterial markers as proxies of dental caries and periodontitis suggests that the observed alcohol-microbiota associations are independent of oral diseases. Lastly, the oral wash sample might not be representative of oral bacterial profiles in specific oral niches, such as the subgingival plaque, which is more directly involved in oral disease.

## Conclusions

We found that alcohol consumption is related to overall oral microbiome community composition and to the abundance of specific oral taxa. Heavy drinking may influence bacterial composition, including potential depletion of beneficial commensal bacteria and increased colonization of potentially pathogenic bacteria. Such changes potentially contribute to alcohol-related diseases, including periodontal disease, head and neck cancer, and digestive tract cancers, but further research is needed to relate alcohol-related composition changes to disease phenotypes. The taxa we have identified can be further investigated to tease out their potential relationship with underlying oral health status and to elucidate their potential role in alcohol-related health consequences. Future studies should also investigate the impact of alcohol drinking on the metagenomic (functional) content of the oral microbiome. Improved understanding of the causes and health impacts of oral dysbiosis can lead to microbiome-targeted approaches for disease prevention.

## Additional file

Additional file 1: **Tables S1-S4** and **Figures S1-S5. Table S1.** Number of sequence reads per sample assigned to reference taxonomy map. **Table S2.** Mean counts of taxa associated with drinking levels in all study participants and subjects in each cohort. **Table S3.** Associations between oral bacteria diversity and abundance of order Lactobacillales, genera Actinomyces and Neisseria with alcohol drinking levels in sensitivity analysis. **Table S4.** Mean counts and fold changes of taxa associated with type of alcoholic beverages in all study participants. **Figure S1.** Beta-diversity of oral bacterial communities in the paired mouthwash-saliva samples. **Figure S2.** Distribution of alcohol consumption in the enterotypes. **Figure S3.** Count boxplots of order Lactobacillales and selected genera that were associated with drinking level. **Figure S4.** Partial Constrained Analysis of Principal Coordinates plots. **Figure S5.** Richness and evenness of oral microbiome by alcohol drinking type. (DOCX 1148 kb)

## Abbreviations

ACS: American Cancer Society
ADH: Alcohol dehydrogenase
CAP: Constrained analysis of principal coordinates
CI: Confidence interval
CPS II: Cancer Prevention Study II
FCs: Fold changes
FDR: False discovery rate
HD: Heavy drinkers
HOMD: Human Oral Microbiome Database
LAB: Lactic acid bacteria
MD: Moderate drinkers
NCI: National Cancer Institute
OTUs: Operational taxonomic units
PCoA: Principal coordinate analysis
PERMANOVA: Permutational Multivariate Analysis of Variance
PLCO: Prostate, Lung, Colorectal, and Ovarian Cancer Screening Trial
WHO: World Health Organization
