# Supplementary material for: Drinking alcohol is associated with variation in the human oral microbiome in a large study of American adults
Source: Microbiome. 2018 Apr 24;6:59. doi: 10.1186/s40168-018-0448-x (PMC5914044; doi:10.1186/s40168-018-0448-x)
Supplement: Supplementary file 1 — Tables S1-S4 and Figures S1-S5. Table S1. Number of sequence reads per sample assigned to reference taxonomy map. Table S2. Mean counts of taxa associated with drinking levels in all study participants and subjects in each cohort. Table S3. Associations between oral bacteria diversity and abundance of order Lactobacillales, genera Actinomyces and Neisseria with alcohol drinking levels in sensitivity analysis. Table S4. Mean counts and fold changes of taxa associated with type of alcoholic beverages in all study participants. Figure S1. Beta-diversity of oral bacterial communities in the paired mouthwash-saliva samples. Figure S2. Distribution of alcohol consumption in the enterotypes. Figure S3. Count boxplots of order Lactobacillales and selected genera that were associated with drinking level. Figure S4. Partial Constrained Analysis of Principal Coordinates plots. Figure S5. Richness and evenness of oral microbiome by alcohol drinking type. (DOCX 1148 kb) [file 40168_2018_448_MOESM1_ESM.docx]

**Table S1: Number of sequence reads per sample assigned to reference taxonomy map.**

| **Cohort** | **Minimum** | **Maximum** | **Mean** | **SD** |
| --- | --- | --- | --- | --- |
| CPSII-a | 6,074 | 26,680 | 9,833 | 3,206 |
| CPSII-b | 3,032 | 17,850 | 9,645 | 2,249 |
| PLCO-a | 1,352 | 28,580 | 8,589 | 3,087 |
| PLCO-b | 4,940 | 26,280 | 11,590 | 1,644 |
| Total | 1,352 | 28,580 | 10,100 | 2,721 |

* Sequence reads per sample which were assigned to taxa by using the Human Oral Microbiome Database (HOMD) pre-defined taxonomy map of reference sequences with ≥98% identity

**Figure S1: Beta-diversity of oral bacterial communities in the paired mouthwash-saliva samples**

**
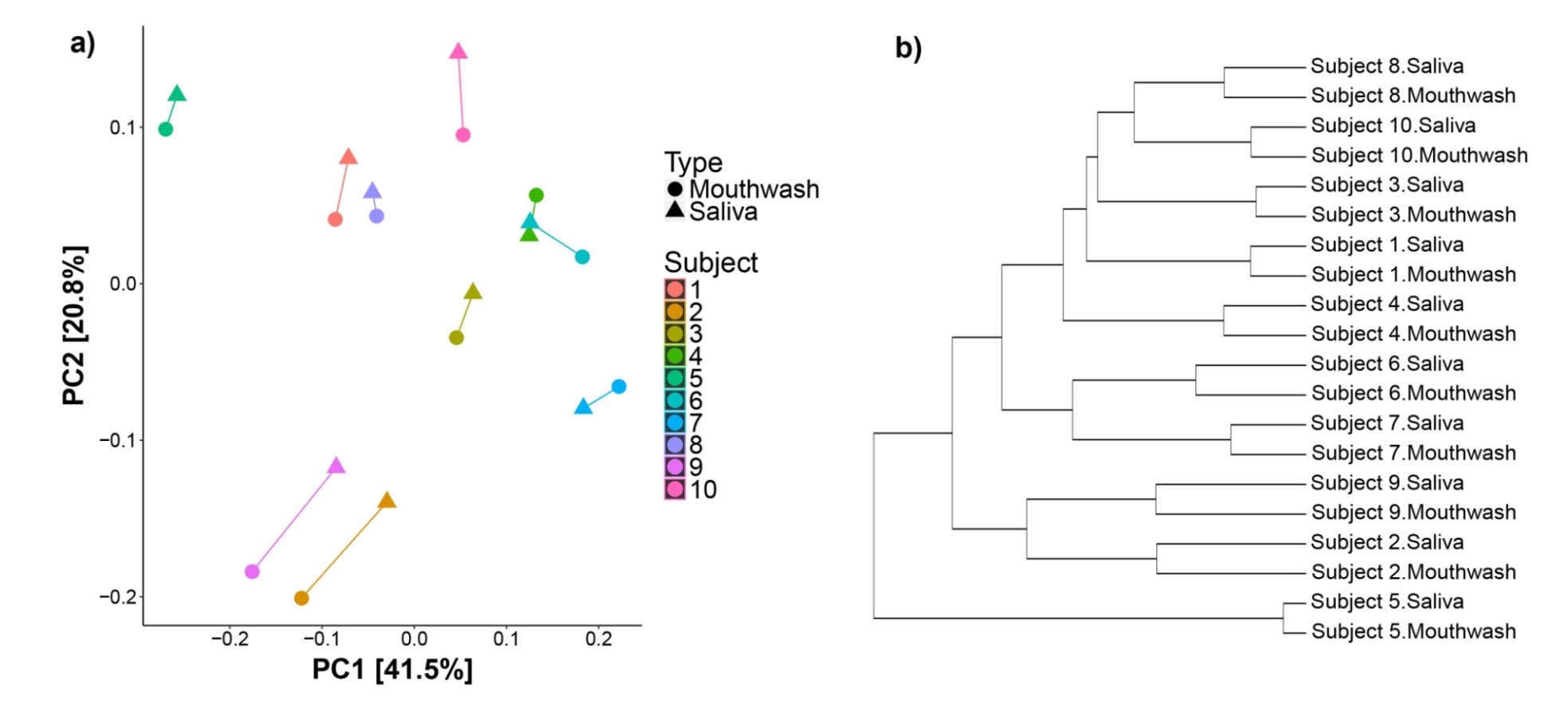
**

We collected paired “Scope” mouthwash samples and saliva samples from 10 healthy subjects. Whole saliva was collected by using spitting method: saliva was allowed to accumulate in the floor of the mouth and the subject spitted it out into a specimen tube every 60 seconds [[1](#_ENREF_1)]. After saliva sample collection, subject was asked to swish vigorously with 10 mL Scope mouthwash (P&G) for 30 seconds, and then to expectorate into another specimen tube. Both saliva and mouthwash samples were transported to laboratory and stored at -80°C within 5 minutes after collection. Illumina MiSeq 16S rRNA gene sequencing was performed for all samples. Principal coordinate analysis (PCoA) (a) and hierarchical cluster analysis (b) representation of JSD distances shows that paired samples clustered together. The results indicate that the within-subject variabilities is less than between-subject variabilities among the paired mouthwash-saliva samples.

**Figure S2: Distribution of alcohol consumption in the enterotypes**

**
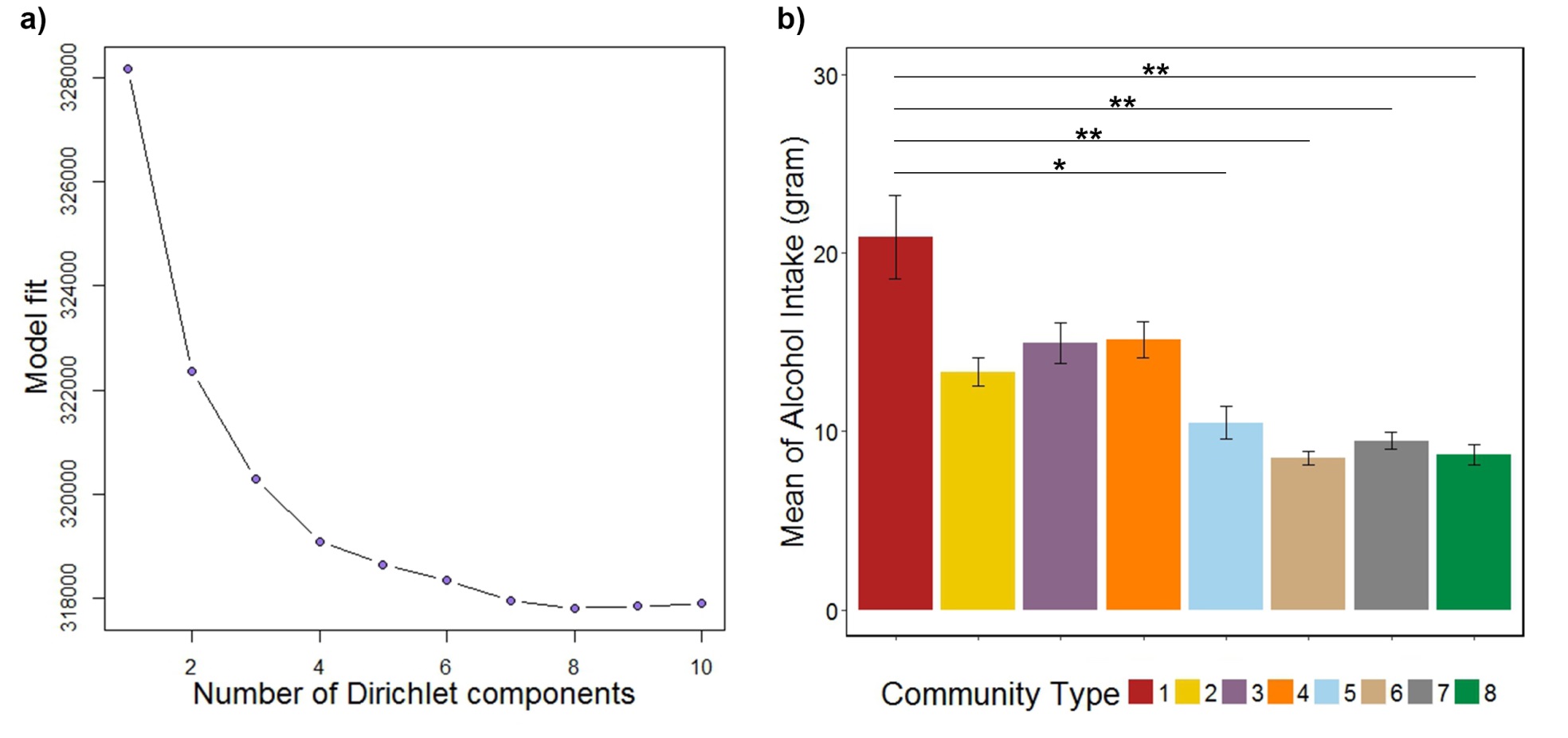
**

a) Fitting to the Dirichlet multinomial mixture (DMM) model indicates optimal classification for all oral samples into eight community types. b) The mean values of ethanol intake (gram per day) in each community type were compared using general linear model. Community type 5 - 8 had significant decreased ethanol intake compared to community type 1. One star (*) indicates p value < 0.05, and two stars (**) indicates p < 0.01.

**Figure S3: Count boxplots of order *Lactobacillales* and selected genera that were associated with drinking level**

**
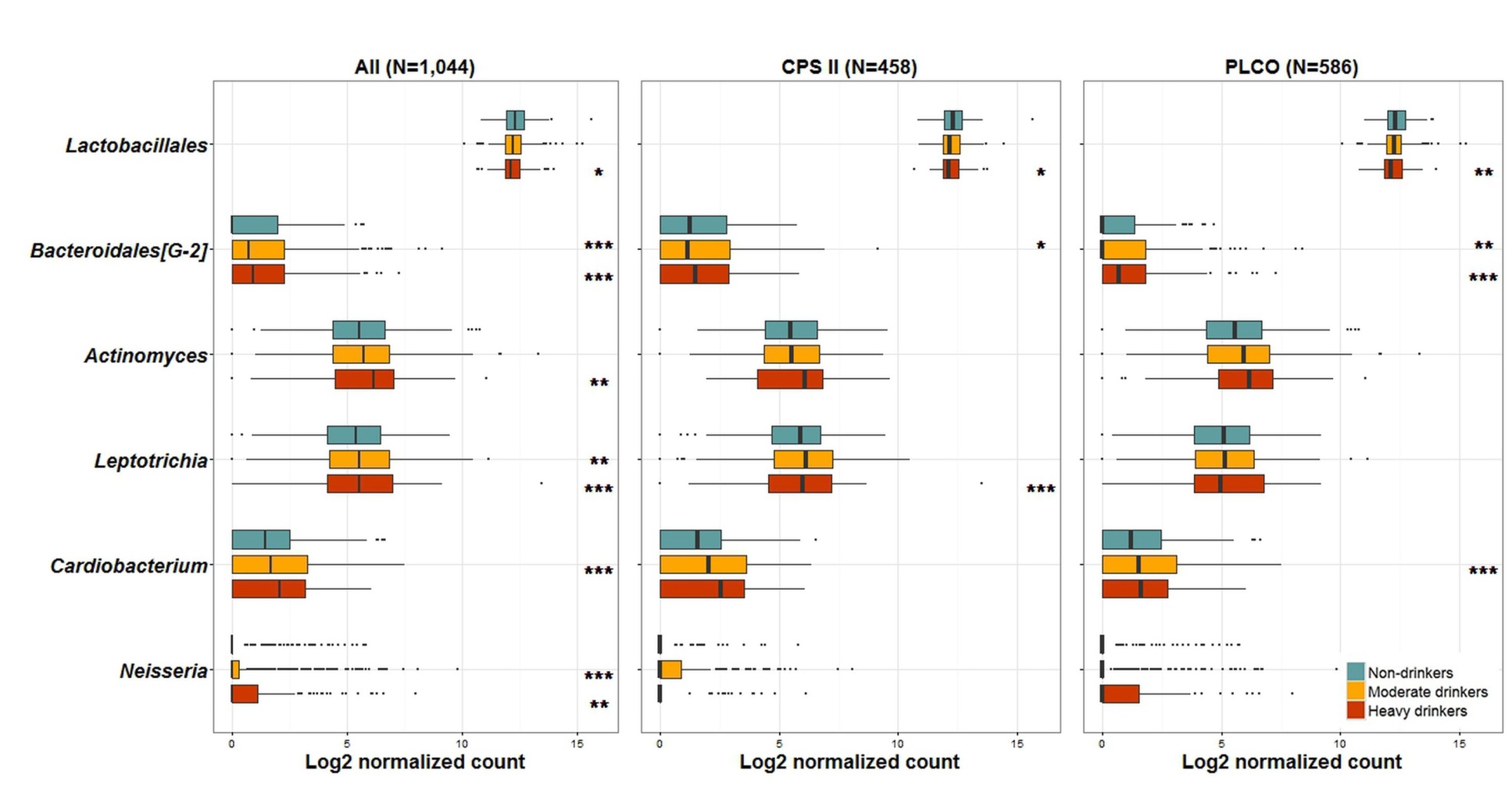
**Counts in each drinking level were plotted for all participants, and in the CPS II and PLCO cohorts separately. All counts were normalized for DESeq2 size factors and log2 transformed after adding a pseudocount of 1. One star (*) indicates q < 0.10, two stars (**) indicates q < 0.05, three stars (***) indicates q < 0.01, compared to non-drinkers in the DESeq2 analysis.

**Figure S4: Partial Constrained Analysis of Principal Coordinates (CAP) plots**

(a-d) Partial CAP plots using unweighted and weighted UniFrac phylogenetic distance matrices for participants in the CPS II cohort and PLCO cohort separately. Drinking level was the constraining variable; age, race, gender, BMI, study, education, smoking status, and study were treated as partial variables. Triangle indicates non-drinkers, square indicates moderate drinkers, and diamond indicates heavy drinkers; filled shapes indicate centroids for each group.


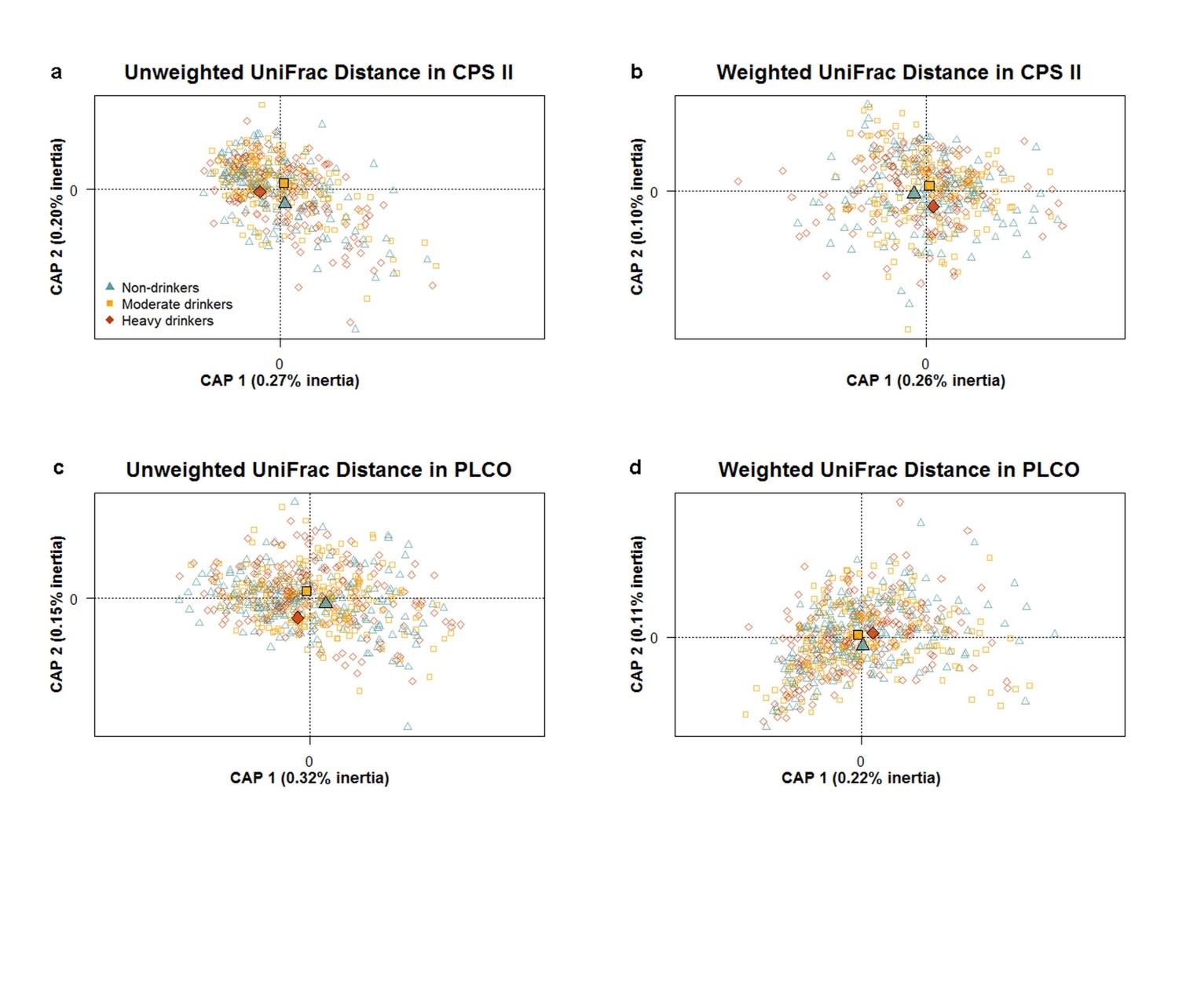


**Table S2: Mean counts of taxa* associated with drinking levels in all study participants and subjects in each cohort**

|  | | **Mean counts**^†^ | | |  |
| --- | --- | --- | --- | --- | --- |
|  | | **All**  **(n=1,044)** | **CPS II**  **(n=458)** | **PLCO**  **(n=586)** | **p for Heterogeneity test**^\|\|^ |
| CLASS | |  |  |  |  |
| *Firmicutes;Bacilli* | |  |  |  |  |
|  | Non-drinkers | 5986.51 | 6055.26 | 5934.73 |  |
|  | Moderate-drinkers | 5578.79 | 5425.16 | 5698.58 |  |
|  | Heavy-drinkers | 5459.46 | 5481.38 | 5441.07 |  |
|  | p^‡^ | 0.0055 | 0.054 | 0.093 | 0.47 |
|  | q^§^ | 0.050 |  |  |  |
| ORDER | |  |  |  |  |
| *Firmicutes;Bacilli;*  *Lactobacillales* | |  |  |  |  |
|  | Non-drinkers | 5687.89 | 5817.08 | 5590.59 |  |
|  | Moderate-drinkers | 5340.36 | 5213.42 | 5439.34 |  |
|  | Heavy-drinkers | 5111.72 | 5115.52 | 5108.53 |  |
|  | p | 0.0050 | 0.031 | 0.11 | 0.64 |
|  | q | 0.064 |  |  |  |
| *Proteobacteria;Grammaproteobacteria;*  *Cardiobacteriales* | |  |  |  |  |
|  | Non-drinkers | 5.24 | 5.74 | 4.86 |  |
|  | Moderate-drinkers | 6.81 | 8.51 | 6.26 |  |
|  | Heavy-drinkers | 6.84 | 9.67 | 4.47 |  |
|  | p | 0.0092 | 0.024 | 0.15 | 0.99 |
|  | q | 0.083 |  |  |  |
| FAMILY | |  |  |  |  |
| *Bacteroidetes;Bacteroides;*  *Bacteroidales;Bacteroidales[F-2]* | |  |  |  |  |
|  | Non-drinkers | 3.28 | 5.34 | 1.72 |  |
|  | Moderate-drinkers | 6.21 | 8.50 | 4.42 |  |
|  | Heavy-drinkers | 6.84 | 7.72 | 6.09 |  |
|  | p | <0.0001 | 0.21 | <0.0001 | 0.019 |
|  | q | 0.0020 |  |  |  |
| *Actinobacteria;Actinobacteria;*  *Actinomycetales;Corynebacteriaceae* | |  |  |  |  |
|  | Non-drinkers | 22.72 | 27.83 | 45.27 |  |
|  | Moderate-drinkers | 29.62 | 35.96 | 49.39 |  |
|  | Heavy-drinkers | 30.79 | 31.47 | 33.99 |  |
|  | p | 0.0050 | 0.34 | 0.0019 | 0.094 |
|  | q | 0.083 |  |  |  |
| *Proteobacteria;Grammaproteobacteria;*  *Pasteurellales;Pasteurellaceae* | |  |  |  |  |
|  | Non-drinkers | 138.73 | 193.68 | 97.34 |  |
|  | Moderate-drinkers | 194.42 | 283.35 | 125.08 |  |
|  | Heavy-drinkers | 191.54 | 259.36 | 134.64 |  |
|  | p | 0.0064 | 0.15 | 0.016 | 0.41 |
|  | q | 0.052 |  |  |  |
| *Proteobacteria;Grammaproteobacteria;*  *Cardiobacteriales;Cardiobacteriaceae* | |  |  |  |  |
|  | Non-drinkers | 5.69 | 6.37 | 5.17 |  |
|  | Moderate-drinkers | 7.56 | 8.37 | 6.92 |  |
|  | Heavy-drinkers | 7.48 | 10.77 | 4.73 |  |
|  | p | 0.0071 | 0.027 | 0.10 | 0.84 |
|  | q | 0.0083 |  |  |  |
| GENUS | |  |  |  |  |
| *Fimicutes;Clostridia;*  *Clostridiales;Lachnospiraceae[14];*  *Lachnoanaerobaculum_gid_046* | |  |  |  |  |
|  | Non-drinkers | 16.10 | 16.18 | 16.04 |  |
|  | Moderate-drinkers | 16.45 | 15.08 | 17.53 |  |
|  | Heavy-drinkers | 25.01 | 23.01 | 26.69 |  |
|  | p | 0.0063 | 0.24 | 0.0034 | 0.24 |
|  | q | 0.042 |  |  |  |
| *Firmicutes;Bacilli;*  *Lactobacillales;Streptococcaceae;*  *Streptococcaus_gid_304* | |  |  |  |  |
|  | Non-drinkers | 37.39 | 50.16 | 27.78 |  |
|  | Moderate-drinkers | 181.57 | 88.92 | 253.80 |  |
|  | Heavy-drinkers | 58.55 | 105.76 | 18.93 |  |
|  | p | <0.001 | 0.041 | 0.59 | 0.33 |
|  | q | 0.015 |  |  |  |
| *Bacteroidetes;Bacteroides;*  *Bacteroidales;Bacteroidales[F-2];*  *Bacteroidales[G-2]* | |  |  |  |  |
|  | Non-drinkers | 3.31 | 5.36 | 1.75 |  |
|  | Moderate-drinkers | 7.05 | 9.62 | 5.05 |  |
|  | Heavy-drinkers | 6.94 | 6.88 | 6.98 |  |
|  | p | <0.0001 | 0.26 | <0.0001 | 0.025 |
|  | q | 0.0027 |  |  |  |
| *Bacteroidetes;Bacteroides;*  *Bacteroidales;Porphyromonadaceae;*  *Tannerella_gid_164* | |  |  |  |  |
|  | Non-drinkers | 2.28 | 2.84 | 1.86 |  |
|  | Moderate-drinkers | 3.00 | 3.56 | 2.57 |  |
|  | Heavy-drinkers | 3.61 | 4.00 | 3.28 |  |
|  | p | 0.0070 | 0.13 | 0.024 | 0.55 |
|  | q | 0.042 |  |  |  |
| *Bacteroidetes;Bacteroides;*  *Bacteroidales;Prevotellaceae;*  *Prevotella_gid_168* | |  |  |  |  |
|  | Non-drinkers | 1.73 | 1.65 | 1.80 |  |
|  | Moderate-drinkers | 43.72 | 2.05 | 76.21 |  |
|  | Heavy-drinkers | 1.98 | 2.41 | 1.63 |  |
|  | p | <0.001 | 0.21 | 0.0099 | 0.22 |
|  | q | 0.0099 |  |  |  |
| *Bacteroidetes;Flavobacterial;*  *Flavobacteriales;Flavobacteriaceae;*  *Bergeyella* | |  |  |  |  |
|  | Non-drinkers | 3.62 | 4.92 | 2.64 |  |
|  | Moderate-drinkers | 4.62 | 6.08 | 3.48 |  |
|  | Heavy-drinkers | 5.29 | 6.83 | 4.00 |  |
|  | p | <0.001 | 0.072 | 0.0036 | 0.45 |
|  | q | 0.014 |  |  |  |
| *Actinobacteria;Actinobacteria;*  *Actinomycetale;Actinomycetaceae;*  *Actinomyces* | |  |  |  |  |
|  | Non-drinkers | 103.79 | 81.51 | 120.58 |  |
|  | Moderate-drinkers | 123.63 | 80.61 | 157.18 |  |
|  | Heavy-drinkers | 131.63 | 110.49 | 149.37 |  |
|  | p | 0.0047 | 0.18 | 0.083 | 0.72 |
|  | q | 0.041 |  |  |  |
| *Actinobacteria;Actinobacteria;*  *Actinomycetales;Corynebacteriaceae;*  *Corynebacterium* | |  |  |  |  |
|  | Non-drinkers | 21.93 | 28.69 | 16.84 |  |
|  | Moderate-drinkers | 28.95 | 35.36 | 23.95 |  |
|  | Heavy-drinkers | 29.51 | 29.51 | 29.50 |  |
|  | p | 0.0064 | 0.66 | <0.001 | 0.029 |
|  | q | 0.042 |  |  |  |
| *Fusobacteria;Fusobacteria;*  *Fusobacteriales;Fusobacteriaceae;*  *Fusobacterium_gid_114* | |  |  |  |  |
|  | Non-drinkers | 184.52 | 282.81 | 110.48 |  |
|  | Moderate-drinkers | 369.96 | 281.27 | 439.12 |  |
|  | Heavy-drinkers | 229.27 | 356.32 | 122.67 |  |
|  | p | 0.018 | 0.25 | 0.030 | 0.39 |
|  | q | 0.090 |  |  |  |
| *Fusobacteria;Fusobacteria;*  *Fusobacteriales;Leptotrichiaceae;*  *Leptotrichia* | |  |  |  |  |
|  | Non-drinkers | 70.25 | 85.20 | 58.99 |  |
|  | Moderate-drinkers | 94.78 | 124.41 | 71.67 |  |
|  | Heavy-drinkers | 157.05 | 249.53 | 79.45 |  |
|  | p | <0.0001 | <0.0001 | 0.072 | 0.097 |
|  | q | 0.0027 |  |  |  |
| *Fusobacteria;Fusobacteria;*  *Fusobacteriales;Leptotrichiaceae;*  *Leptotrichia_gid_126* | |  |  |  |  |
|  | Non-drinkers | 7.97 | 9.71 | 6.65 |  |
|  | Moderate-drinkers | 10.31 | 12.97 | 8.23 |  |
|  | Heavy-drinkers | 12.10 | 11.14 | 12.91 |  |
|  | p | 0.010 | 0.32 | 0.0033 | 0.14 |
|  | q | 0.055 |  |  |  |
| *Proteobacteria;Gammaproteobacteria;*  *Pasteurellales;Pasteurellaceae;*  *Aggregatibacter_gid_286* | |  |  |  |  |
|  | Non-drinkers | 3.21 | 3.07 | 3.32 |  |
|  | Moderate-drinkers | 10.94 | 16.66 | 6.49 |  |
|  | Heavy-drinkers | 5.63 | 6.62 | 4.81 |  |
|  | p | 0.0024 | 0.088 | 0.067 | 0.71 |
|  | q | 0.027 |  |  |  |
| *Proteobacteria;Betaproteobacteria;*  *Neisseriales;Neisseriaceae;*  *Eikenella* | |  |  |  |  |
|  | Non-drinkers | 1.33 | 1.62 | 1.11 |  |
|  | Moderate-drinkers | 2.20 | 2.73 | 1.78 |  |
|  | Heavy-drinkers | 1.75 | 2.15 | 1.42 |  |
|  | p | 0.0099 | 0.080 | 0.055 | 0.82 |
|  | q | 0.054 |  |  |  |
| *Proteobacteria;Betaproteobacteria;*  *Neisseriales;Neisseriaceae;*  *Kingella_gid_009* | |  |  |  |  |
|  | Non-drinkers | 4.19 | 4.77 | 3.75 |  |
|  | Moderate-drinkers | 8.35 | 12.40 | 5.18 |  |
|  | Heavy-drinkers | 6.12 | 4.84 | 7.20 |  |
|  | p | 0.0057 | 0.32 | 0.0039 | 0.15 |
|  | q | 0.042 |  |  |  |
| *Proteobacteria;Grammaproteobacteria;*  *Cardiobacteriales;Cardiobacteriaceae;*  *Cardiobacterium* | |  |  |  |  |
|  | Non-drinkers | 5.41 | 6.15 | 4.85 |  |
|  | Moderate-drinkers | 7.47 | 7.98 | 7.08 |  |
|  | Heavy-drinkers | 7.04 | 9.84 | 4.68 |  |
|  | p | 0.0047 | 0.055 | 0.043 | 0.87 |
|  | q | 0.041 |  |  |  |
| *Proteobacteria;Betaproteobacteria;*  *Neisseriales;Neisseriaceae;*  *Neisseria_gid_382* | |  |  |  |  |
|  | Non-drinkers | 1.90 | 1.20 | 2.42 |  |
|  | Moderate-drinkers | 5.20 | 4.09 | 6.06 |  |
|  | Heavy-drinkers | 5.55 | 2.59 | 8.04 |  |
|  | p | 0.0047 | 0.14 | 0.012 | 0.25 |
|  | q | 0.041 |  |  |  |
| SPECIES | |  |  |  |  |
| *Firmicutes;Bacilli;*  *Lactobacillales;Carnobacteriaceae;*  *Granulicatella;adiacens_gid_296* | |  |  |  |  |
|  | Non-drinkers | 133.08 | 128.79 | 136.32 |  |
|  | Moderate-drinkers | 110.70 | 107.74 | 113.00 |  |
|  | Heavy-drinkers | 105.93 | 115.16 | 98.09 |  |
|  | p | <0.001 | 0.25 | 0.0026 | 0.23 |
|  | q | 0.057 |  |  |  |
| *Firmicutes;Bacilli;*  *Lactobacillales;Carnobacteriaceae;*  *Granulicatella;Elegans_gid_337* | |  |  |  |  |
|  | Non-drinkers | 15.86 | 6.46 | 22.94 |  |
|  | Moderate-drinkers | 23.52 | 14.07 | 30.89 |  |
|  | Heavy-drinkers | 22.88 | 22.28 | 23.38 |  |
|  | p | 0.0013 | <0.001 | 0.23 | 0.036 |
|  | q^†^ | 0.057 |  |  |  |
| *Actinobacteria;Actinobacteria;*  *Actinomycetale;Actinomycetaceae;*  *Actinomyces;Graevenitzii_gid_484* | |  |  |  |  |
|  | Non-drinkers | 37.25 | 23.01 | 47.97 |  |
|  | Moderate-drinkers | 47.59 | 22.78 | 66.93 |  |
|  | Heavy-drinkers | 55.77 | 34.46 | 73.65 |  |
|  | p | 0.0030 | 0.11 | 0.024 | 0.82 |
|  | q | 0.088 |  |  |  |
| *Bacteroidetes;Bacteroides;*  *Bacteroidales;Bacteroidales [F-2];*  *Bacteroidales[G-2];Sp.oral taxon 274_gid_156* | |  |  |  |  |
|  | Non-drinkers | 1.64 | 2.45 | 1.04 |  |
|  | Moderate-drinkers | 2.43 | 3.02 | 1.97 |  |
|  | Heavy-drinkers | 2.59 | 2.84 | 2.38 |  |
|  | p | 0.0043 | 0.40 | 0.0051 | 0.23 |
|  | q | 0.094 |  |  |  |

* The association between alcohol drinking level and abundance of taxa was detected by DESeq function, adjusted for age, sex, race, BMI, smoking status, education, and study.

^†^ Sequence read counts were normalized for DESeq2 size factors.

^‡^ Nominal p-values from trend tests in all study participants, or in each cohort. To test for trend, alcohol drinking was treated as a continuous variable by assigning the numbers 0, 1, and 2 to non-, moderate-, and heavy-drinkers, respectively. All taxa with an FDR-adjusted q<0.10 are included in the table.

^§^  FDR-adjusted p-value. FDR adjustment was conducted at each level (i.e. class, genus) separately.

^||^ p-values for heterogeneity were calculated from Chi-square test of Cochran’s Q statistic.

**Table S3: Associations between oral bacteria diversity and abundance of order *Lactobacillales*, genera *Actinomyces* and *Neisseria* with alcohol drinking levels in sensitivity analysis**

|  |  | **Richness*** | | **Beta-diversity**^†^ | ***Lactobacillales***^‡^ | | ***Actinomyces***^‡^ | | ***Neisseria***^‡^ | |
| --- | --- | --- | --- | --- | --- | --- | --- | --- | --- | --- |
|  |  | Median | *p* | *p* | Mean counts | *p* trend | Mean counts | *p* trend | Mean counts | *p* trend |
| **Excluding current smokers (n)** | |  |  |  |  |  |  |  |  |  |
|  | Heavy drinker (136) | 103.02 | 0.0070 | 0.0032 | 5108.57 |  | 130.60 |  | 6.25 |  |
|  | Moderate drinker (567) | 100.92 | 0.0160 | 0.3200 | 5337.53 |  | 116.64 |  | 5.45 |  |
|  | Non-drinker (249) | 96.56 |  |  | 5463.04 | 0.0559 | 89.74 | 0.0004 | 2.05 | 0.0031 |
| **Excluding *P.g* and/or *A.a* carriers**^§^ | |  |  |  |  |  |  |  |  |  |
|  | Heavy drinker (105) | 95.15 | 0.0390 | 0.0190 | 4974.37 |  | 153.57 |  | 5.58 |  |
|  | Moderate drinker (427) | 95.69 | 0.0740 | 0.6500 | 5433.57 |  | 131.65 |  | 3.76 |  |
|  | Non-drinker (185) | 91.32 |  |  | 5893.19 | 0.0018 | 115.07 | 0.0025 | 1.55 | 0.0047 |
| **Low *S.mutans* (below median)** | |  |  |  |  |  |  |  |  |  |
|  | Heavy drinker (95) | 102.06 | 0.0310 | 0.0640 | 4919.13 |  | 140.20 |  | 6.98 |  |
|  | Moderate drinker (301) | 100.84 | 0.1560 | 0.2500 | 4954.27 |  | 157.70 |  | 8.80 |  |
|  | Non-drinker (126) | 96.41 |  |  | 5267.54 | 0.0459 | 111.55 | 0.0370 | 2.15 | 0.0760 |
| **High *S.mutans* (above median)** | |  |  |  |  |  |  |  |  |  |
|  | Heavy drinker (65) | 103.28 | 0.0690 | 0.0980 | 5276.79 |  | 111.17 |  | 2.75 |  |
|  | Moderate drinker (313) | 99.95 | 0.0270 | 0.2660 | 5866.38 |  | 92.06 |  | 2.24 |  |
|  | Non-drinker (144) | 95.38 |  |  | 6455.58 | 0.0117 | 86.38 | 0.3900 | 1.90 | 0.1700 |

* Richness was calculated in 500 iterations of rarefied OTU tables with the minimum sequencing depth of 1,325 among all study subjects; linear regression with covariates adjustment was used to examine the difference of richness among drinking groups.

^†^ Permutational Multivariate Analysis of Variance (PERMANOVA) according to unweighted UniFrac distance.

^‡^ The association of the abundance of order *Lactobacillales*, genera *Actinomyces* and *Neisseria* with alcohol drinking was detected by DESeq function, adjusted for covariates; sequence read counts were normalized by dividing raw counts by DESeq size factors**.**

^§^ Subject carried species *Porphyromonas gingivalis* and/or *Aggregatibacter actinomycetemcomitans*.

**Figure S5: Richness and evenness of oral microbiome by alcohol drinking type**

(a-b) Violin plots of number of observed species (richness) and Inverse Simpson’s Index (evenness) in non-drinker, wine only drinker (n=101), beer only drinker (n=39), and liquor only drinker (n=26) groups. These indices were calculated for 500 iterations of rarefied OTU table with minimum sequencing depth of 1,325 among all study subjects, and the average over the iterations was taken for each participant. Plotted are median, interquartile ranges, and the probability density of the indices at different values. Mean values of the richness in non-, wine-, beer-, liquor- drinkers were 94.6, 100.9, 88.1 and 91.6; mean values of the evenness in each group were 11.6, 12.6, 11.7, and 11.0. (c-d) Rarefaction curves of number of observed species and Inverse Simpson’s Index according to the number of reads per sample in non-drinker, wine only drinker, beer only drinker, and liquor only drinker groups.


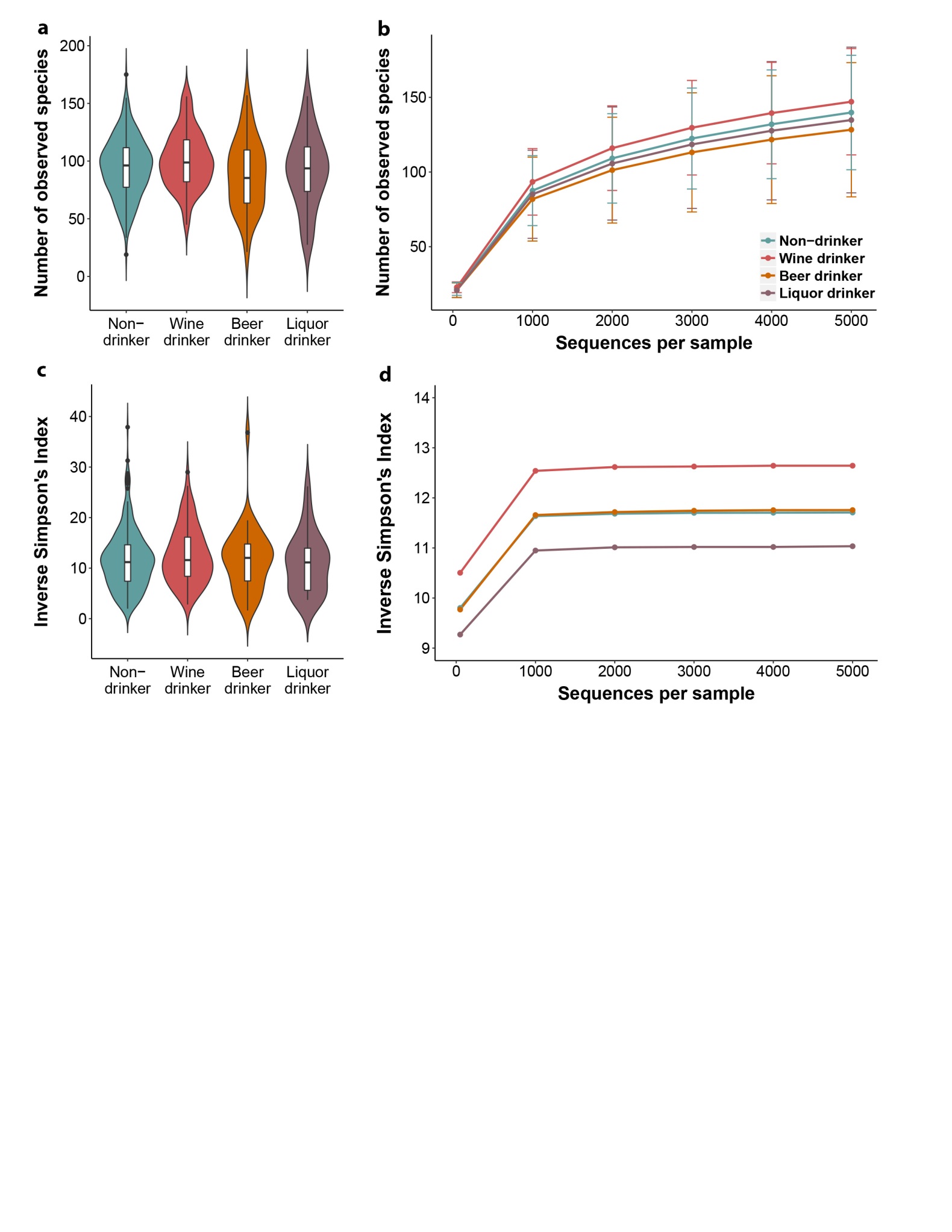


**Table S4: Mean counts and fold changes of taxa*** **associated with type of alcoholic beverages in all study participants**

|  | | **Mean counts**^†^ | **Fold change** | **q**^‡^ |
| --- | --- | --- | --- | --- |
| PHYLUM | |  |  |  |
| *Firmicutes* | |  |  |  |
|  | Non-drinkers (n=270) | 7082.32 |  |  |
|  | Wine drinkers (n=101) | 6155.02 | 0.90 (0.82, 0.98) | 0.074 |
|  | Beer drinkers (n=39) | 9189.07 | 1.13 (1.01, 1.26) | 0.086 |
|  | Liquor drinkers (n=26) | 6918.56 | 0.92 (0.82, 1.04) | 0.47 |
| *Bacteroidetes* | |  |  |  |
|  | Non-drinkers | 1201.28 |  |  |
|  | Wine drinkers | 991.58 | 0.82 (0.69, 0.98) | 0.074 |
|  | Beer drinkers | 1026.41 | 0.77 (0.61, 0.96) | 0.086 |
|  | Liquor drinkers | 1142.05 | 0.85 (0.67, 1.07) | 0.47 |
| CLASS | |  |  |  |
| *Firmicutes;Clostridia* | |  |  |  |
|  | Non-drinkers | 1015.30 |  |  |
|  | Wine drinkers | 954.46 | 0.90 (0.78, 1.04) | 0.32 |
|  | Beer drinkers | 775.45 | 0.78 (0.65, 0.93) | 0.055 |
|  | Liquor drinkers | 889.71 | 0.86 (0.71, 1.05) | 0.60 |
| ORDER | |  |  |  |
| *Firmicutes;Clostridia;Clostridiales* | |  |  |  |
|  | Non-drinkers | 1040.01 |  |  |
|  | Wine drinkers | 982.62 | 0.89 (0.77, 1.04) | 0.32 |
|  | Beer drinkers | 807.03 | 0.77 (0.63, 0.93) | 0.081 |
|  | Liquor drinkers | 890.72 | 0.85 (0.69, 1.03) | 0.48 |
| FAMILY | |  |  |  |
| *Firmicutes;Clostridia;Clostridiales;*  *Peptococcaceae* | |  |  |  |
|  | Non-drinkers | 1.98 |  |  |
|  | Wine drinkers | 0.86 | 0.44 (0.25, 0.78) | 0.066 |
|  | Beer drinkers | 1.34 | 0.63 (0.30, 1.33) | 0.46 |
|  | Liquor drinkers | 2.11 | 0.85 (0.39, 1.85) | 0.90 |
| *Actinobacteria;Actinobacteria;Actinomycetales;*  *Corynebacteriaceae* | |  |  |  |
|  | Non-drinkers | 22.72 |  |  |
|  | Wine drinkers | 32.59 | 1.55 (1.17, 2.05) | 0.053 |
|  | Beer drinkers | 21.51 | 1.39 (0.97, 1.99) | 0.32 |
|  | Liquor drinkers | 19.88 | 1.51 (1.03, 2.21) | 0.43 |
| GENUS | |  |  |  |
| *Firmicutes;Clostridia;Clostridiales;*  *Lachnospiraceae[14];Lachnospiraceae[G-2]* | |  |  |  |
|  | Non-drinkers | 24.94 |  |  |
|  | Wine drinkers | 11.80 | 0.57 (0.36, 0.89) | 0.12 |
|  | Beer drinkers | 18.34 | 0.67 (0.37, 1.19) | 0.37 |
|  | Liquor drinkers | 6.99 | 0.39 (0.21, 0.71) | 0.090 |
| *Firmicutes;Clostridia;Clostridiales;*  *Peptostreptococcaceae[11];Mogibacterium_gid_007* | |  |  |  |
|  | Non-drinkers | 16.08 |  |  |
|  | Wine drinkers | 10.91 | 0.66 (0.50, 0.88) | 0.071 |
|  | Beer drinkers | 10.85 | 0.75 (0.52, 1.09) | 0.37 |
|  | Liquor drinkers | 14.64 | 0.86 (0.58, 1.28) | 0.78 |
| *Firmicutes;Clostridia;Clostridiales;*  *Lachnospiraceae[14];Oribacterium* | |  |  |  |
|  | Non-drinkers | 52.26 |  |  |
|  | Wine drinkers | 40.68 | 0.92 (0.71, 1.19) | 0.66 |
|  | Beer drinkers | 22.13 | 0.62 (0.44, 0.87) | 0.094 |
|  | Liquor drinkers | 48.83 | 0.95 (0.67, 1.36) | 0.90 |
| *Firmicutes;Clostridia;Clostridiales;*  *Peptococcaceae;Peptococcus* | |  |  |  |
|  | Non-drinkers | 1.89 |  |  |
|  | Wine drinkers | 0.79 | 0.45 (0.26, 0.80) | 0.073 |
|  | Beer drinkers | 1.08 | 0.65 (0.31, 1.36) | 0.43 |
|  | Liquor drinkers | 1.80 | 0.85 (0.39, 1.83) | 0.82 |
| *Firmicutes;Clostridia;Clostridiales;*  *Lachnospiraceae[14];Stomatobaculum_gid_237* | |  |  |  |
|  | Non-drinkers | 26.52 |  |  |
|  | Wine drinkers | 21.46 | 0.86 (0.62, 1.19) | 0.57 |
|  | Beer drinkers | 23.21 | 0.56 (0.37, 0.86) | 0.098 |
|  | Liquor drinkers | 12.25 | 0.65 (0.41, 1.02) | 0.33 |
| *Bacteroidetes;Bacteroides;Bacteroidales;*  *Porphyromonadaceae;Porphyromonas_gid_155* | |  |  |  |
|  | Non-drinkers | 13.12 |  |  |
|  | Wine drinkers | 12.27 | 0.80 (0.44, 1.44) | 0.67 |
|  | Beer drinkers | 3.39 | 0.32 (0.15, 0.68) | 0.071 |
|  | Liquor drinkers | 2.54 | 0.43 (0.19, 0.96) | 0.26 |
| *Bacteroidetes;Bacteroides;Bacteroidales;*  *Prevotellaceae;Prevotella_gid_170* | |  |  |  |
|  | Non-drinkers | 378.84 |  |  |
|  | Wine drinkers | 244.68 | 0.58 (0.43, 0.77) | 0.016 |
|  | Beer drinkers | 194.76 | 0.51 (0.35, 0.74) | 0.016 |
|  | Liquor drinkers | 170.80 | 0.39 (0.26, 0.58) | <0.001 |
| *Actinobacteria;Actinobacteria;Actinomycetales;*  *Corynebacteriaceae;Corynebacterium* | |  |  |  |
|  | Non-drinkers | 21.93 |  |  |
|  | Wine drinkers | 32.81 | 1.59 (1.21, 2.09) | 0.022 |
|  | Beer drinkers | 20.40 | 1.45 (1.02, 2.07) | 0.21 |
|  | Liquor drinkers | 18.93 | 1.60 (1.10, 2.33) | 0.19 |
| *Actinobacteria;Actinobacteria;Bifidobacteriales;*  *Bifidobacteriaceae;Parascardovia* | |  |  |  |
|  | Non-drinkers | 2.20 |  |  |
|  | Wine drinkers | 2.36 | 0.78 (0.37, 1.65) | 0.66 |
|  | Beer drinkers | 28.38 | 5.87 (2.23, 15.47) | 0.016 |
|  | Liquor drinkers | 0.58 | 0.95 (0.34, 2.68) | 0.95 |
| *Proteobacteria;Gammaproteobacteria;Pasteurellales;*  *Pasteurellaceae;Aggregatibacter_gid_286* | |  |  |  |
|  | Non-drinkers | 3.21 |  |  |
|  | Wine drinkers | 5.32 | 2.50 (1.32, 4.72) | 0.071 |
|  | Beer drinkers | 21.29 | 3.05 (1.34, 6.94) | 0.098 |
|  | Liquor drinkers | 3.66 | 2.03 (0.84, 4.87) | 0.45 |
| *Proteobacteria;Betaproteobacteria;Neisseriales;*  *Neisseriaceae;Eikenella* | |  |  |  |
|  | Non-drinkers | 1.33 |  |  |
|  | Wine drinkers | 2.41 | 1.86 (1.29, 2.69) | 0.022 |
|  | Beer drinkers | 2.03 | 1.36 (0.84, 2.21) | 0.39 |
|  | Liquor drinkers | 2.94 | 1.86 (1.12, 3.09) | 0.19 |

* The association between alcohol drinking type and taxon abundance was detected by DESeq function, adjusted for age, sex, race, BMI, smoking status, education, drinking amount, and study. For each drinking type, other types of beverages and the cross-product term were adjusted in the model. All taxa with an FDR-adjusted q<0.10 are included in the table.

^†^ Counts were normalized by dividing raw counts by DESeq size factors**.**

^‡^ FDR-adjusted p-value. FDR adjustment was conducted at each level (i.e. class, genus) separately.

**References**

1. Navazesh M: **Methods for collecting saliva**. *Annals of the New York Academy of Sciences* 1993, **694**:72-77.
